# Human gut microbiota–reactive DP8α Tregs prevent acute graft-versus-host disease in a CD73-dependent manner

**DOI:** 10.1172/jci.insight.179458

**Published:** 2024-08-01

**Authors:** Emmanuelle Godefroy, Patrice Chevallier, Fabienne Haspot, Caroline Vignes, Véronique Daguin, Sylvia Lambot, Margaux Verdon, Margaux De Seilhac, Valentin Letailleur, Anne Jarry, Annabelle Pédron, Thierry Guillaume, Pierre Peterlin, Alice Garnier, Marie-Anne Vibet, Maxence Mougon, Amandine Le Bourgeois, Maxime Jullien, Francine Jotereau, Frédéric Altare

**Affiliations:** 1 Nantes Université, Univ Angers, INSERM, CNRS, Immunology and New Concepts in ImmunoTherapy, INCIT,UMR 1302, F-44000 Nantes, France.; 2 LabEx IGO, Nantes University, Nantes, France.; 3 CHU de Nantes, F-44000 Nantes, France.; 4 INSERM UMR 1307, CRCI2NA IRS-UN, Nantes Université, Nantes, France.; 5 Nantes Université, INSERM, Center for Research in Transplantation and Translational Immunology, UMR 1064, F-44000 Nantes, France.; 6 Maat Pharma, Lyon, France.; 7 Université Libre de Bruxelles, Institute for Medical Immunology, and ULB Center for Research in Immunology, Gosselies, Belgium.; 8 Department of Biostatistics, Centre Hospitalier Universitaire (CHU) de Nantes, Nantes, France.

**Keywords:** Immunology, Transplantation, Immunotherapy, T cells, Translation

## Abstract

Graft-versus-host disease (GvHD) is a life-threatening complication frequently occurring following allogeneic hematopoietic stem cell transplantation (allo-HSCT). Since gut microbiota and regulatory T cells (Tregs) are believed to play roles in GvHD prevention, we investigated whether DP8α Tregs, which we have previously described to harbor a T cell receptor specificity for the gut commensal *Faecalibacterium prausnitzii*, could protect against GvHD, thereby linking the microbiota and its effect on GvHD. We observed a decrease in CD73^+^ DP8α Treg frequency in allo-HSCT patients 1 month after transplantation, which was associated with acute GvHD (aGvHD) development at 1 month after transplantation, as compared with aGvHD-free patients, without being correlated to hematological disease relapse. Importantly, CD73 activity was shown to be critical for DP8α Treg suppressive function. Moreover, the frequency of host-reactive DP8α Tregs was also lower in aGvHD patients, as compared with aGvHD-free patients, which could embody a protective mechanism responsible for the maintenance of this cell subset in GvHD-free patients. We also showed that human DP8α Tregs protected mice against xenogeneic GvHD through limiting deleterious inflammation and preserving gut integrity. Altogether, these results demonstrated that human DP8α Tregs mediate aGvHD prevention in a CD73-dependent manner, likely through host reactivity, advocating for the use of these cells for the development of innovative therapeutic strategies to preclude aGvHD-related inflammation.

## Abstract 2

Human microbiota-reactive regulatory T cells, so-called DP8a, are associated with acute graft-versus-host disease (GvHD) protection in patients treated through allogeneic hematopoietic stem cell transplantation and prevent it in a xeno-GvHD mouse model.

## Graphical abstract

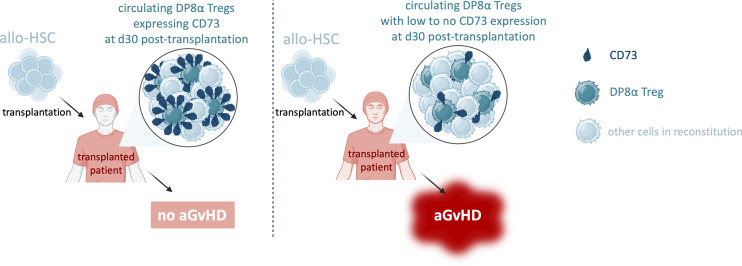

## Introduction

A number of hematological disorders are currently treated with myeloablative or reduced-intensity conditioning regimens, followed by infusion of allogeneic hematopoietic stem cell transplantation (allo-HSCT) (1). Alloreactive donors’ T cells contained in the graft can induce graft-versus-host disease (GvHD), a potentially deadly complication whereby graft-derived cells attack healthy host tissues. Acute GvHD (aGvHD) and chronic GvHD (cGvHD) occur in 30%–50% and 30%–70% of allo-HSCT patients, respectively (2–5). While steroids remain the first-line treatment for GvHD, new treatments are needed to prevent/improve this condition, avoid steroid complications, and overcome corticosteroid resistance.

As such, regulatory T cells (Tregs) able to inhibit alloreactivity represent potential attractive targets to treat and predict GvHD. Indeed, several studies demonstrated the protective role of these cells against GvHD in mice (6–9), while preserving graft-versus-tumor activity (8). In humans, grafts containing donor-derived Tregs have been shown to represent a promising strategy (10). Moreover, a change in the adenosine/purinergic pathway, particularly on the surface of Tregs, seems to be involved in GvHD development (11, 12). This pathway hydrolyzes proinflammatory ATP into immunosuppressive adenosine, mainly through both CD39 and CD73 enzymatic activities. Gut bacteria can also promote adenosine production or secrete inosine, an adenosine metabolite that also triggers adenosine receptors, highlighting part of the complex host/microbiota interplay in the regulation of this purinergic pathway (13).

Intestinal microbiota and its α diversity at engraftment has been shown to represent an independent biomarker to predict mortality, particularly regarding death due to transplantation-related causes, in allo-HSCT patients (14). Increasing evidences also indicate that gut microbiota influences GvHD outcome (15). Among these, general loss of diversity, particularly observed upon broad-spectrum antibiotic use (16–20) and decreases in the relative abundance of several classes of Clostridia, such as *Blautia* or *Faecalibacterium*, have been linked to GvHD severity (20–24). Accordingly, several clinical studies demonstrated the efficacy of fecal microbiota transplantation in steroid-resistant GvHD patients (25–27), although without identifying the underlying mechanisms. Moreover, mice gavaged with Treg-inducing Clostridia displayed milder GvHD and improved survival (28), suggesting that microbiotherapy could rely on microbiota-mediated Treg induction. Yet, no clear mechanism linking gut microbiota and the adenosine/purinergic pathway of Tregs in the control of GvHD has been unraveled.

In this context, we have identified, in human colon and blood, a FoxP3-negative and IL-10–secreting Treg subset, coexpressing CD4 and CD8α together with CCR6 and CXCR6, hence named DP8α Tregs (29, 30), displaying a T cell receptor (TCR) specificity for the gut commensal *Faecalibacterium prausnitzii* (29–31), a Clostridium IV member. These cells might thus represent the human counterpart of mouse FoxP3^+^ Tregs induced by Clostridia (32), as both populations protect against colitis in vivo and share the master transcription factor RORγt (32–34). Importantly, DP8α Tregs also express high levels of CD39 and CD73, which drive their suppressive function (31).

For these reasons, we hypothesized that *F*. *prausnitzii*–reactive DP8α Tregs could bridge microbiota dysbiosis and GvHD occurrence, and therefore investigated their role in GvHD development.

## Results

### GvHD patients display a decrease in CD73^+^ DP8α Treg frequency 1 month after transplantation.

To characterize the DP8α Treg subset in allo-HSCT patients, we screened a cohort of 62 consecutive patients, allografted for various hematological diseases in Nantes University Hospital’s Hematology Department between October 2018 and December 2020. Twenty-five patients developed grade 2–4 aGvHD, according to Mount Sinai International Consortium grading (35), at a median of 28 days after transplantation, ranging from 10 to 112 days (see Table 1 for patients’ information).

**Figure 1:**
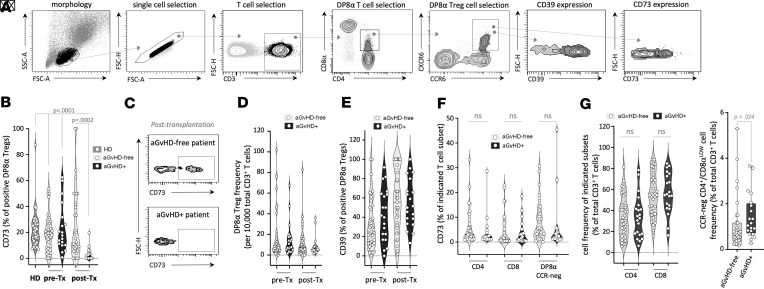
CD73 expression and, to a lower extent, DP8α Treg frequency, is specifically decreased in aGvHD patients 1 month after transplantation. (**A**) The gating strategy to study DP8α Tregs is shown. (**B** and **G**) Blood samples from healthy donors (HD) and from patients with hematological malignancies were collected before receiving allo-HSCT (pre-Tx, d–7) and at 1 month after transplantation (post-Tx). Samples were analyzed by flow cytometry to assess CD3^+^, CD4^+^, CD8α^lo^, CCR6^+^, and CXCR6^+^ DP8α Treg–related characteristics, including their CD73 expression pattern shown here at indicated time points. All patients tested (**B**) and representative examples at d30 (**C**) are shown. One-way ANOVA (Kruskal-Wallis test) followed by Dunn’s multiple-comparison test to obtain adjusted *P* values was used. (**D**) DP8α Treg frequency among total CD3^+^ T cells is shown at indicated time points. (**E**) CD39 expression on DP8α Tregs from allo-HSCT patients is shown at indicated time points. (**F**) CD73 expression, on d30 after transplantation, by indicated T cell subset: total single-positive (SP) CD4^+^ T cells, total SP CD8^+^ T cells, and DP8α non-Treg cells, annotated DP8α CCR-neg (i.e., DP8α cells comprising CCR6^–^CXCR6^–^, CCR6^+^CXCR6^–^, and CCR6^–^CXCR6^+^ fractions) is shown. Mann-Whitney tests were performed for single comparisons. (**G**) Cell frequencies of indicated subsets on d30 after transplantation in aGvHD patients are shown. Mann-Whitney tests were performed for single comparisons.

Blood samples were collected from patients before (d–7) and after allo-HSCT (d30) and PBMCs analyzed by flow cytometry to determine DP8α Treg frequency among total T cells, but also among CD4^+^ and CD8^+^ T cells, given that these 2 subsets do not reconstitute evenly. Additionally, CD39 and CD73 expression patterns by DP8α Tregs, particularly elevated on this subset (31), were monitored (see Figure 1A for gating strategy) as a proxy for DP8α Treg suppressive potential since we had previously observed that blocking this purinergic pathway, at the early CD39-dependent step, abolished DP8α regulatory activity (31). Remarkably, aGvHD development was strongly associated with a lack of CD73 expression on DP8α Tregs (mean = 3% ± 1.1%), on d30 after transplantation (Figure 1, B and C), as compared with aGvHD-free patients (mean = 25% ± 4.7%; *P* = 0.0002), but also compared with patients before transplantation who will eventually develop aGvHD (mean = 23% ± 4.2%; *P* < 0.0001), or not (mean = 19% ± 2.3%; *P* < 0.0001), as well as with healthy individuals (*n* = 38; mean = 23% ± 2.3%; *P* < 0.0001). No difference was observed before transplantation between aGvHD and aGvHD-free patients (Figure 1B). DP8α Treg frequency also tended to decrease after transplantation in aGvHD-positive patients (*n* = 21; mean = 0.0029% ± 0.00114%) versus aGvHD-free patients (*n* = 32; mean = 0.039% ± 0.0180%) among total CD3^+^ (Figure 1D), CD4^+^, or CD8^+^ T cells (data not shown). In contrast, their CD39 expression was statistically comparable between patients who developed aGvHD and those who did not and was elevated, as a mean, in all groups (Figure 1E).

Of note, no other T cell subset displayed similar changes, including total CD4^+^ and CD8^+^ T cells and DP8α cells lacking CCR6 and/or CXCR6 expression (Figure 1, F and G), which do not respond to *F*. *prausnitzii* (31).

**Figure 2:**
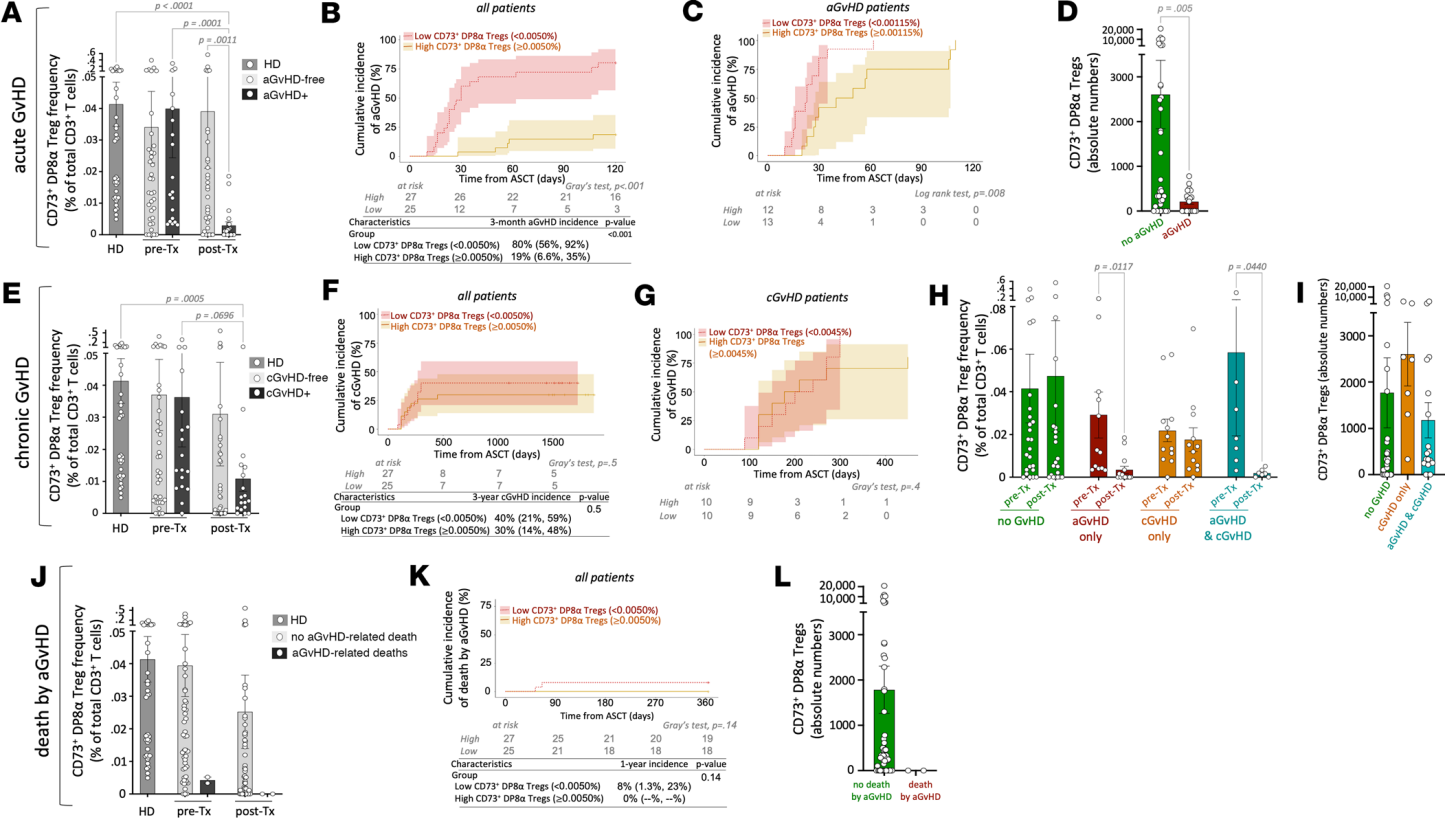
CD73^+^ DP8α Treg abundance is associated with aGvHD occurrence and severity, but not with cGvHD. (**A**) CD73^+^ DP8α Treg frequencies are shown in HD and allo-HSCT patients, both before and after transplantation in aGvHD and aGvHD-free patients. One-way ANOVA (Kruskal-Wallis tests) followed by Dunn’s multiple-comparison test to obtain adjusted *P* values was used. Results are represented as mean ± SEM. (**B**) Cumulative aGvHD incidence over time was plotted for low versus high CD73^+^ DP8α Treg abundance. Cutoff was determined using the median of CD73^+^ DP8α Treg frequency among total T cells (= 0.0050%) from all patients. The Fine-Gray method, with relapse or death as competing risks, was used (shaded area: 95% CI). (**C**) Cumulative aGvHD incidence over time was plotted for low versus high CD73^+^ DP8α Treg abundance in the aGvHD-positive subgroup, using the median of this group of patients (= 0.00115%) as a cutoff. Log-rank (Mantel-Cox) test was used; shaded area is 95% CI. (**D**) Absolute numbers of CD73^+^ DP8α Tregs in 30 mL samples in aGvHD versus aGvHD-free patients were calculated using CBC clinical data. Mann-Whitney test was used to compare both groups (mean ± SEM). (**E**) CD73^+^ DP8α Treg frequencies are shown in HD and allo-HSCT patients, in cGvHD and cGvHD-free patients (1-way ANOVA [Kruskal-Wallis test] followed by Dunn’s multiple-comparison test, mean ± SEM). (**F**) Cumulative cGvHD incidence, over approximatively 1500 days (corresponding to the 52-week median follow-up for this cohort, calculated using reverse Kaplan-Meier), was plotted for low versus high CD73^+^ DP8α Treg abundance in all patients (same cutoff as above, Fine-Gray test with relapse or death as competing risks; shaded area is 95% CI). (**G**) Cumulative cGvHD incidence was plotted for low versus high CD73^+^ DP8α Treg abundance in the cGvHD-positive subgroup, using the median of this group of patients (= 0.0045%) as a cutoff (Fine-Gray test with relapse or death as competing risks; shaded area is 95% CI). (**H**) CD73^+^ DP8α Treg frequencies are shown in indicated patients’ groups (1-way ANOVA). (**I**) Absolute numbers of CD73^+^ DP8α Tregs in 30 mL samples in cGvHD versus aGvHD-free patients. Mann-Whitney test was used to compare both groups (mean ± SEM). (**J**) CD73^+^ DP8α Treg frequencies are shown in HD and allo-HSCT patients, in patients who died of GvHD or not (1-way ANOVA [Kruskal-Wallis test] followed by Dunn’s multiple-comparison test, mean ± SEM). (**K**) Cumulative death by aGvHD incidence was plotted for low versus high CD73^+^ DP8α Treg abundance in all patients (same cutoff as above, Fine-Gray test with death unrelated to aGVHD as a competing risk). (**L**) Absolute numbers of CD73^+^ DP8α Tregs in 30 mL samples in patients who died of aGvHD or not. Mann-Whitney test was used to compare both groups (mean ± SEM).

As CD73 expression 1 month after transplantation (Figure 1, B and C) on DP8α Tregs and, to a lower extent, the frequency of these cells among total T cells (Figure 1D), were both specifically decreased in aGvHD, as compared with aGvHD-free patients, we therefore chose to take these 2 parameters into account and thereby followed the frequency of CD73^+^ DP8α Tregs among total CD3^+^ T cells to track the variations of these Tregs in GvHD versus GvHD-free patients. CD73^+^ DP8α Treg mean frequency on d30 after transplantation was drastically lower in aGvHD patients (mean = 0.0029% ± 0.00114%), as compared with aGvHD-free patients (mean = 0.039% ± 0.0180%; *P* = 0.0011), but also compared with patients before transplantation who will eventually develop aGvHD (mean = 0.0399% ± 0.0156%; *P* = 0.0001), or with healthy individuals (*n* = 38; mean = 0.0413% ± 0.0069%; *P* < 0.0001) (Figure 2A). Moreover, the median of CD73^+^ DP8α Treg frequency 1 month after transplantation in the entire cohort, i.e., 0.0050% of total T cells, was used to stratify patients into low versus high CD73^+^ DP8α Treg abundance. Cumulative aGvHD incidence was strikingly higher in the low arm than the in the high one (Figure 2B; Fine-Gray test, *P* < 0.001), with a hazard ratio of 7.74 (95% CI: 3.356 to 17.83), strongly supporting the notion that CD73^+^ DP8α Tregs protect against aGvHD. Additionally, an analysis of the cumulative incidence of aGvHD in the subgroup of patients who developed the disorder showed that those with lower levels of CD73^+^ DP8α Tregs experienced significantly earlier onset (log-rank test, *P* = 0.008; Figure 2C). Furthermore, aGvHD patients had lower absolute numbers of CD73^+^ DP8α Tregs than aGvHD-free patients did (Figure 2D).

Notably, upon aGvHD diagnosis, patients received corticotherapy (prednisone tablets, Cortancyl or methylprednisolone, i.v., MePRD, Solu-Medrol, obtained from Nantes University Hospital, Hematology Department, Nantes, France). Importantly, the decrease in the frequency of CD73^+^ DP8α Tregs did not result from this corticotherapy, since it was equally observed in patients already under treatment or not on d30 after transplantation sampling (Supplemental Figure 1A; supplemental material available online with this article; https://doi.org/10.1172/jci.insight.179458DS1). Of note, methylprednisolone exposure in aGvHD patients who received it before the 1-month sampling lasted for 24.4 ± 2.7 days as a mean, ranging from 1 to 31 days (median: 27 days). Furthermore, in vitro MePRD treatment of healthy donor–derived PBMCs did not change CD73 expression on any T cell subset tested (data not shown), including DP8α Tregs (Supplemental Figure 1B).

Since all patients were transplanted more than 3 years prior to this study, we could also study whether CD73^+^ DP8α Treg frequency was also associated with the risk of cGvHD. A trend similar to that for aGvHD was observed, although not statistically significant; i.e., CD73^+^ DP8α Treg frequency among total T cells 1 month after transplantation was lower in cGvHD^+^ patients (mean = 0.011% ± 0.004%) than in either cGvHD-free patients (mean = 0.031% ± 0.016%; *P* < 0.07) or heathy individuals (*n* = 38; mean = 0.0413% ± 0.0069%; *P* = 0.0005) (Figure 2E). CD73^+^ DP8α Treg frequency before transplantation was similar in both patients groups as well as comparable to levels observed in healthy individuals (Figure 2E). Then, patients were stratified into low versus high CD73^+^ DP8α Treg abundance, at 1 month after transplantation, as above. Cumulative cGvHD incidence was not statistically different between both arms (Figure 2F; Fine-Gray test, *P* > 0.5), suggesting that CD73^+^ DP8α Tregs have no effect on cGvHD. Among cGvHD patients only, the cumulative cGvHD incidence over time was analogous in high versus low CD73^+^ DP8α Treg arms, advocating once again for a lack of influence of these cells at 1 month after transplantation on cGvHD development (Figure 2G, *P* > 0.4). Interestingly, among patients who developed cGvHD, but not aGvHD, no significant difference was observed between their CD73^+^ DP8α Treg levels 1 month after transplantation and GvHD-free patients or cGvHD patients before transplantation (Figure 2H). Seven patients underwent both aGvHD and cGvHD, explaining the seemingly lower CD73^+^ DP8α Treg frequency when all cGvHD patients were considered, irrespective of their aGvHD history. Additionally, no significant difference in absolute numbers of CD73^+^ DP8α Tregs was observed between GvHD-free patients and cGvHD patients, regardless of their aGvHD status (Figure 2I). Altogether, these data advocate for a lack of a role for DP8α Tregs in cGvHD incidence at 1 month after transplantation.

To start investigating whether low levels of CD73^+^ DP8α Tregs also correlated with aGvHD severity, we compared their frequency with death caused by aGvHD. CD73^+^ DP8α Treg frequency also tended to inversely correlate with death due to aGvHD, as patients who died of aGvHD had lower frequencies of these cells, as compared with all other patients, both before transplantation (mean = 0.004% ± 0.0009% and 0.039% ± 0.0095%, respectively) and after transplantation (d30; mean = 0% ± 0% and 0.0252% ± 0.0095%, respectively) (Figure 2J). Moreover, no aGvHD-related death was observed when CD73^+^ DP8α Treg frequency was above 0.0050%, the global median (Figure 2, J and K). Furthermore, absolute numbers of CD73^+^ DP8α Tregs were lower in patients who died of aGvHD than in patients who did not (Figure 2L). These data strongly suggest that CD73^+^ DP8α Tregs could play a role in aGvHD severity as well. Of note, the abundance of CD73^+^ DP8α Tregs did not correlate with the cumulative incidence of death from other causes than aGvHD (Fine-Gray test, *P* = 0.5; Supplemental Figure 2). Furthermore, taking into account only patients who have died, CD73^+^ DP8α Tregs also tended to inversely correlate with death by aGvHD, as patients who died of aGvHD had undetectable CD73^+^ DP8α Tregs after transplantation (d30), as compared with patients who died of other causes, such as infections or relapse (*n* = 2, mean = 0% and *n* = 18, mean = 0.023% ± 0.0105%, respectively) (Supplemental Figure 3A). When only patients who developed aGvHD were considered, no CD73^+^ DP8α Tregs were detected in patients who died of aGvHD versus those who survived (*n* = 2, mean = 0.004% ± 0.0009% and *n* = 28, mean = 0.038% ± 0.0107%, respectively) before transplantation, and, to a lower extent, after transplantation (*n* = 2, mean = 0% and *n* = 30, mean = 0.008% ± 0.0025%, respectively) (Supplemental Figure 3B). While more patients would need to be screened, these results nonetheless show that particularly low frequencies of CD73^+^ DP8α Tregs, particularly after transplantation, were not only associated with aGvHD development, but also with death caused by aGvHD.

**Figure 3:**
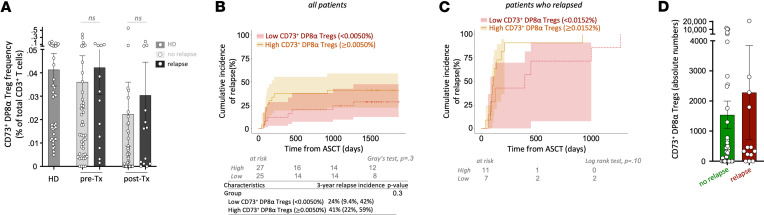
CD73^+^ DP8α Treg frequency does not significantly affect disease relapse. (**A**) CD73^+^ DP8α Treg frequencies are shown in HD and allo-HSCT patients, in patients who relapsed or not (1-way ANOVA [Kruskal-Wallis test] followed by Dunn’s multiple-comparison test, mean ± SEM). (**B**) Cumulative relapse incidence was plotted for low versus high CD73^+^ DP8α Treg abundance in all patients. Cutoff was determined using the median of CD73^+^ DP8α Treg frequency among total T cells (= 0.0050%) from all patients. Fine-Gray test with NRM as a competing risk was used (shaded area: 95% CI). (**C**) Cumulative relapse incidence was plotted for low versus high CD73^+^ DP8α Treg abundance in the relapse subgroup, using the median of this group of patients (= 0.0152%) as a cutoff (Fine-Gray test with NRM as a competing risk was used; shaded area is 95% CI). (**D**) Absolute numbers of CD73^+^ DP8α Tregs in patients who relapsed or not were calculated using CBC clinical data. Mann-Whitney test was used to compare both groups (mean ± SEM).

Next, we wanted to assess whether there was a correlation between relapse and CD73^+^ DP8α Treg frequency. To do so, we first compared relapse incidence in the entire cohort. Although CD73^+^ DP8α Treg frequency appeared slightly higher in patients who relapsed, this difference was not statistically significant, both before and 1 month after transplantation between patients who relapsed (*n* = 12, mean = 0.042% ± 0.0115% and *n* = 13, mean = 0.031% ± 0.0142%, respectively) and patients who did not (*n* = 43, mean = 0.036% ± 0.012% and *n* = 41, mean = 0.022% ± 0.0137%, respectively) (Figure 3A). Analysis of cumulative relapse and non-relapse mortality (NRM) incidence showed no statistical correlation with CD73^+^ DP8α Treg numbers. Three-year relapse and NRM rate were respectively 24% versus 41% (*P* = 0.3) and 24% versus 7.4% (*P* = 0.1), in patients with low versus high numbers of CD73^+^ DP8α Tregs (Figure 3, B and C). Similarly, CD73^+^ DP8α Treg absolute numbers were comparable in patients who relapsed and in patients who did not relapse (Figure 3D). Further studies, including more patients, would therefore be needed to formally determine whether elevated levels of CD73^+^ DP8α Tregs could promote relapse or not.

As antibiotics strongly affect gut microbiota composition, we plotted microbiota-reactive CD73^+^ DP8α Treg frequency according to antibiotic treatment. Unfortunately, many distinct antibiotic molecules were used, preventing the detection of their potential effects on DP8α Tregs (Supplemental Figure 4).

Moreover, in an attempt at assessing the origin of DP8α Tregs analyzed above, we used the chimerism clinical data, which was available for a fraction of the 62 patients (Supplemental Figure 5, A–C). On d30 after transplantation, T cell chimerism was available for 12 patients. Ten patients had 99.6% ± 0.256% T cells of donor origin, while the other 2 patients had low to no donor-derived T cells (Supplemental Figure 5A). Consequently, one could extrapolate that the majority of patients, on d30, had DP8α Tregs of donor origin.

Therefore, low frequencies of CD73^+^ DP8α Tregs during immune reconstitution was strongly associated with aGVHD risk. Multivariate logistic regression analysis confirmed the association between these 2 variables (*P* = 0.0001, OR = 0.07955) (Table 2). Particularly low levels of these cells even appear to increase the risk of dying from GvHD. Other clinical variables, including sex, age, HLA matching, GvHD prophylaxis, and cell dose as well as cGvHD and relapse were analyzed through multivariate logistic regression and showed small association of high CD73^+^ DP8α Tregs with age or reduced-intensity conditioning, but no association was found with sex, HLA matching, GvHD prophylaxis, dose of injected cells, cGvHD, or relapse (Table 2). Altogether, these results thus suggest that these cells could protect against aGvHD, likely through their CD73-mediated suppressive activity, seemingly without significantly increasing the odds of disease relapse.

### DP8α Tregs protect NSG mice against acute xenogeneic GvHD.

In order to determine whether DP8α Tregs could indeed protect against aGvHD, a preclinical in vivo mouse model was undertaken. Unfortunately, a major hurdle to test this function in vivo resides in the fact that no murine Treg counterpart to human DP8α Tregs has yet been formally identified. Consequently, an immunodeficient NSG mouse model was used to test the ability of human DP8α Tregs to protect against human PBMC–induced xenogeneic GvHD (xeno-GvHD).

**Figure 4:**
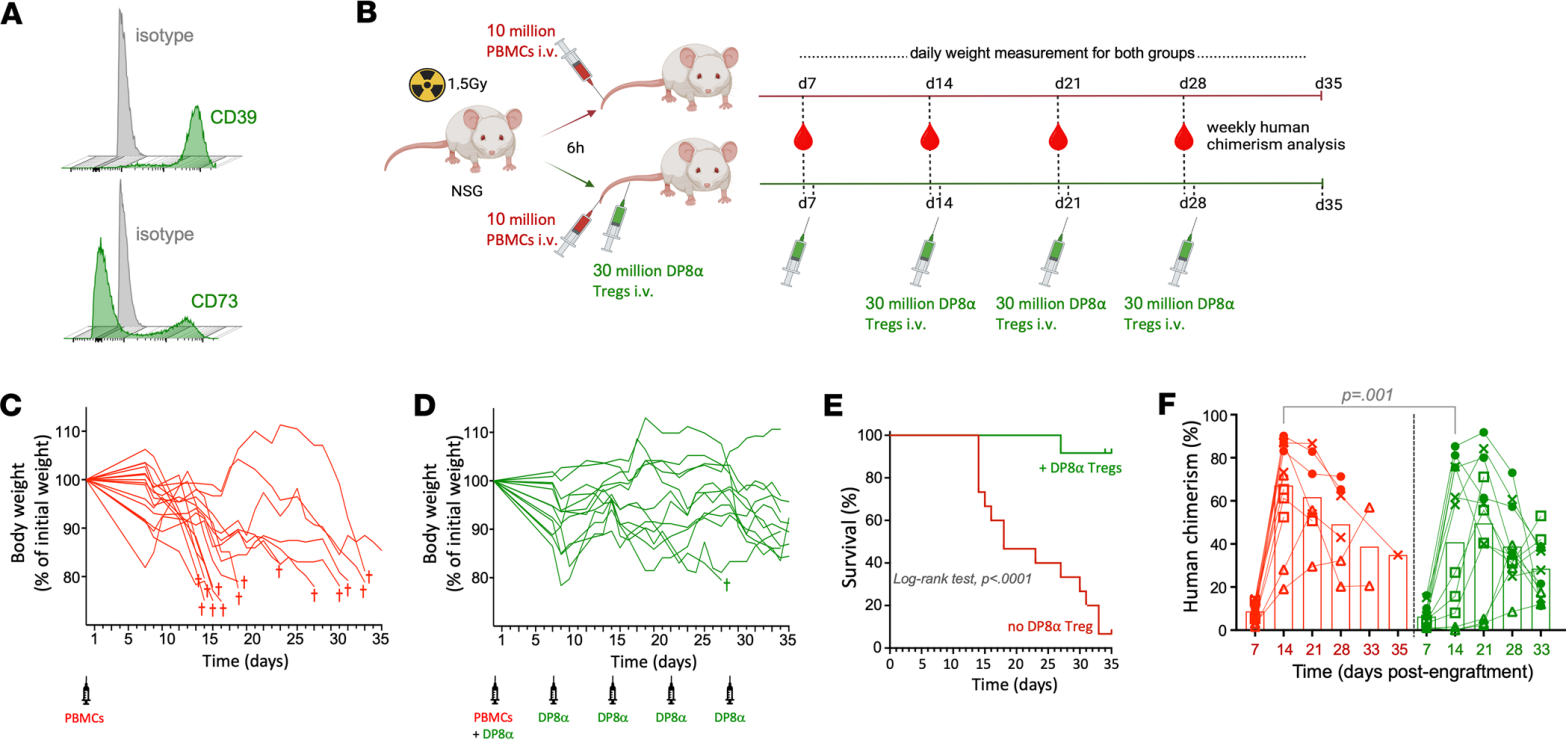
Human DP8α Tregs protect against xeno-GvHD in vivo. (**A**) CD39 and CD73 expression by the DP8α Treg clone used in vivo. (**B**–**D**) NSG mice were irradiated at 1.5 Gy at least 6 hours prior to being i.v. injected with 10 million freshly purified PBMCs from healthy individuals to induce xeno-GvHD (red). Another group of mice was also i.v. injected with 30 million DP8α Treg clonal cells (CD3/CD28-activated 48 hours prior infusion) on d0, d7, d14, d21, and d28 (green). Experiments were repeated using PBMCs from 4 different healthy volunteers with 3 mice per group. (**B**) Schematic describing experiment setup created with BioRender. (**C** and **D**) Mice were weighed almost daily, starting on d7. Mice from the PBMC (**C**) and PBMC+DP8α (**D**) groups had to be sacrificed (†) when they lost 20% of their initial weight. (**E**) Mice survival is shown. Data were analyzed with a log-rank (Mantel-Cox) test. (**F**) Human chimerism was assessed weekly, as described in the Methods section, in the blood of each mouse injected i.v. on d0 with healthy donors’ PBMCs (●, HD1; ×, HD2; Δ, HD3; □, HD4). Wilcoxon’s matched-pairs log-rank test was used.

Following irradiation (1.5 Gy), mice were infused i.v. with human PBMCs alone or together with clonal preactivated DP8α Tregs (30 million), hereafter referred as “PBMC mice” and “PBMC+DP8α mice,” respectively. Of note, DP8α Tregs exhibit their regulatory function once activated. Indeed, we previously showed that IL-10, produced upon activation of the CD39/CD73 pathway, mediated their suppressive activity (30, 31), hence the need for in vitro preactivation prior to their infusion. Importantly, injected DP8α Tregs responded virtually identically whether they were activated with the CD3/CD28 nanomatrix, also used for experiments, or with *F*. *prausnitzii*–derived epitopes presented by antigen-presenting cells (Supplemental Figure 6). The injected DP8α Tregs were shown to coexpress CD39 and CD73 (Figure 4A). Moreover, maintenance of activated Tregs in vivo was obtained through 4 additional weekly injections of in vitro–preactivated DP8α Tregs. The experimental setup is summarized in Figure 4B. Identical experiments were repeated 4 times using PBMCs derived from different donors. The T cell composition of injected PBMCs was similar for the 4 individuals in terms of single-positive CD4^+^, single-positive CD8^+^, DP8α Tregs as well as other double-positive or double-negative subsets (Supplemental Figure 7, A–C), thereby limiting interdonor variation effects. The DP8α Treg fractions corresponded to less than 12 × 10^3^ cells per 10^7^ PBMCs, representing only 0.04% of the 30 × 10^6^ therapeutic DP8α Tregs injected, which one could consider quasi-negligible. Also, PBMC-derived DP8α Tregs not only had comparable frequencies between the 4 individuals, but also all expressed CD39 and CD73 at relatively similar levels (Supplemental Figure 7D).

All mice (*n* = 15) that received solely PBMCs, but one, reached a 20% weight loss from their initial body weight, the major clinical readout for aGvHD development in this model (36, 37), and therefore had to be sacrificed within an 18-day median survival period (Figure 4C). In striking contrast, mice (*n* = 12) that also received DP8α Tregs maintained their overall body weight (Figure 4D). Only one mouse from this latter group was sacrificed before the end of the experiment, on d27 (Figure 4D). Survival curves demonstrated the striking protective effect of DP8α Tregs against acute xeno-GvHD, in this model (Figure 4E).

Blood was collected weekly for each mouse to measure human cell engraftment (Figure 4F). On d7, both groups showed low levels of human chimerism. By d14, the expected disparity of human cell engraftment between each healthy blood donor was similar between both groups (Figure 4F). Human chimerism rapidly expanded within the PBMC mice to reach a plateau on d14, while this plateau was significantly lower and also delayed on d21 in the PBMC+DP8α mice. One can hypothesize that the lower level of chimerism in the PBMC+DP8α group, as compared with the PBMC group, is likely due to the proliferative control exerted by DP8α Tregs on PBMC-derived T cells. Nevertheless, as shown in Figure 4F, all mice from both groups were successfully engrafted.

As previously reported (37, 38), this xeno-GvHD mouse model is virtually entirely T cell dependent. Indeed, on d14, circulating human CD45^+^ (hCD45^+^) cells were largely composed of T cells irrespective of the mouse group, more precisely, greater than 98% as a mean (Supplemental Figure 8, A and B).

Altogether, these data revealed the reproducible ability of DP8α Tregs to prevent weight loss, revealing a radical protective effect of these cells against acute xeno-GvHD in this model.

### DP8α Tregs control systemic inflammation during acute xeno-GvHD.

**Figure 5:**
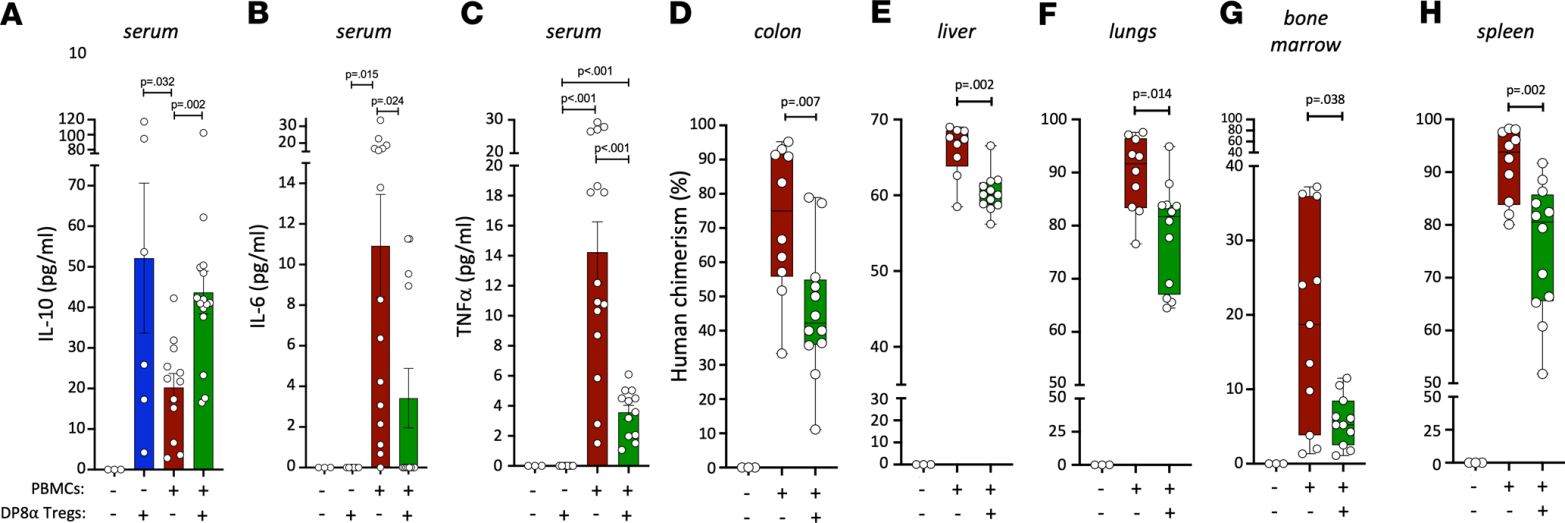
Human DP8α Tregs protect against systemic GvHD-related inflammation in vivo. Clotted blood and organs were harvested at sacrifice. (**A**–**C**) Sera were tested for human IL-10 (**A**), human IL-6 (**B**), and human TNF-α (**C**) cytokines by ELISA. For mice that received either PBMCs only or PBMCs+DP8α Tregs, clotted blood was collected at sacrifice when their weight loss reached 20% of their initial weight, i.e., on day 21.9 ± 2.03 and 34.4 ± 0.61, respectively. The other 2 control groups (naive mice that did not receive any human cells and mice that only received activated DP8α Tregs) were all sacrificed on d14. Results are represented as mean ± SEM. (**D**–**G**) Organs were mashed and filtered before red blood cells were lysed as described in the Methods section. Human chimerism was assessed by flow cytometry in the colon (**D**), liver (**E**), lungs (**F**), bone marrow (**G**), and spleen (**H**) of all animals. One-way ANOVA (Kruskal-Wallis test) followed by Dunn’s multiple-comparison test to obtain adjusted *P* values was used.

To begin investigating the underlying mechanism(s) driving DP8α Treg–mediated protection, we first measured human cytokines in serum at sacrifice. As expected, increased levels of antiinflammatory hIL-10 were detected in PBMC+DP8α mice, as compared with PBMC mice (*P* = 0.002) (Figure 5A), IL-10 being highly produced by DP8α Tregs (30, 31). In parallel, a group of mice (*n* = 6) were injected with a single dose of 30 × 10^6^ preactivated DP8α Treg cells on d0. Fittingly, significant levels of hIL-10 were detected in the serum of the latter mice on d7 (*n* = 3, >50 pg/mL) and still on d14 (*n* = 3, ranging from 4 to 26 pg/mL) (Figure 5A). On the other hand, proinflammatory hTNF-α (*P* < 0.001) and hIL-6 (*P* = 0.024) levels were reduced in PBMC+DP8α mice, as compared with PBMC mice (Figure 5, B and C).

Human cell infiltration in other compartments, namely the colon, liver, lungs, bone marrow (BM), and spleen, was also assessed by flow cytometry after ex vivo dissociation. Human chimerism was lower in all organs from PBMC+DP8α mice, as compared with PBMC mice (Figure 5, D–H).

Therefore, DP8α Treg administration appears to lower inflammation in a systemic manner, in this model.

### DP8α Tregs potently reduce colonic inflammation during acute xeno-GvHD.

Since the colon represents one of the preferential homing sites for alloreactive T cells, making it a target organ of GvHD, as well as a privileged site for DP8α Treg induction/activation, we performed in-depth analyses of T cell infiltrates and of tissue architecture of the colons of mice injected with human PBMCs with or without DP8α Tregs.

To this end, colons were harvested at sacrifice. Flow cytometry data showed that hCD45^+^ cells represented 72% ± 5.2% of total CD45^+^ cells (hCD45^+^ + mCD45^+^) as a mean, in the PBMC group (Figure 5D). In the PBMC+DP8α group, percentages of hCD45^+^ cells were significantly lower (mean = 46% ± 4.8%; *P* = 0.007) (Figure 5D), suggesting a control of hCD45^+^ cells (presumably xeno-reactive T cells) by DP8α Tregs. As previously described (37, 38), this xeno-GvHD model is T cell mediated; thus, as expected, colonic human infiltrates were mostly composed of T cells, as over 96% of human cells expressed hCD3 (Supplemental Figure 9).

**Figure 6:**
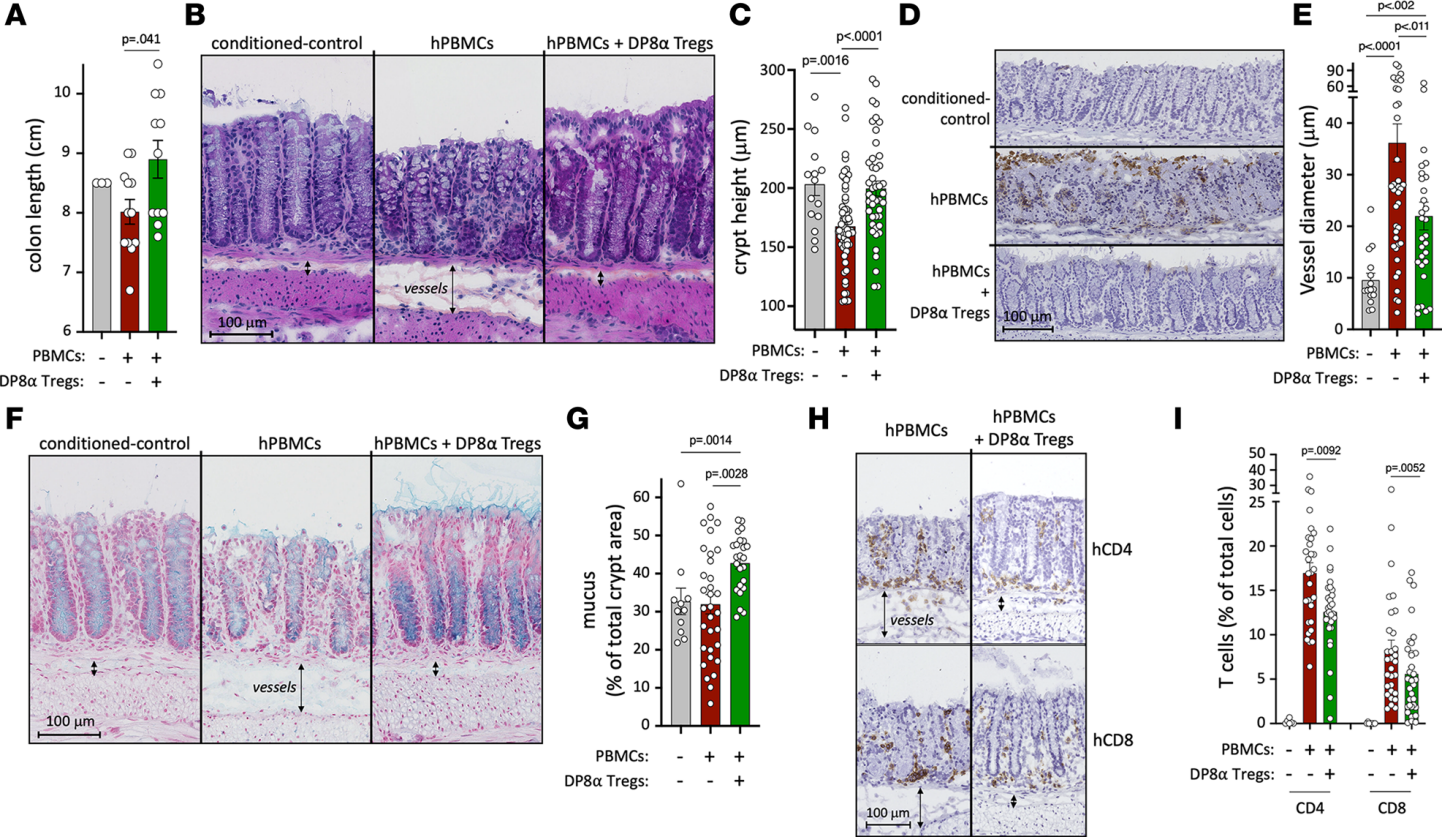
Human DP8α Tregs protect against colonic xeno-GvHD–related inflammation in vivo. Colons were harvested at sacrifice and measured. Colonic segments were split into 2 for mechanical dissociation and subsequent flow cytometry analyses on the one hand, or histological analyses on the other hand. (**A**) Colon length measured at sacrifice. (**B**) Hematoxylin/phloxine/saffron (HPS) staining performed on 5-μm cryostat section of colon for conditioned-control mice and both treated groups. Vessels are also highlighted with arrows. (**C**) Crypt height was measured on 5 representative colonic regions from each mouse on sections harboring correctly oriented villi. (**D**) Apoptosis detection was assessed by immunohistochemistry labeling of activated caspase-3. Representative images are shown for each group of mice. (**E**) Vessel diameter measurements (5 measurements per animal), as highlighted in **C**. (**F**) Alcian blue staining obtained for each group of mice. Vessels are also highlighted with arrows. (**G**) Blue-stained areas, corresponding to secreted mucus and goblet cells producing mucus, and total crypt areas were detected using automatic structure recognition by deep learning in QuPath software. Data are expressed as the percentage of mucus area among the total crypt area from 4 representative regions from each mouse. (**H**) Immunostainings of human CD4 or CD8α for both groups. (**I**) Percentages of positive cells for CD4 or CD8α expression among the total number of cells in colonic mucosa. The percentage of positive cells was determined using the positive cell detection function of QuPath from 5 regions of interest for each mouse. Scale bars: 100 μm.

Additionally, colons from PBMC mice were significantly shorter (mean = 8 ± 0.21 cm, *P* = 0.041) than those from PBMC+DP8α mice (mean = 8.9 ± 0.32 cm) and tended to also be shorter than those from irradiated-only (referred as “conditioned”) NSG mice (mean = 8.5 ± 0.60 cm) at sacrifice (Figure 6A), strongly suggesting that DP8α Tregs can curb colonic inflammation.

Next, histological analyses revealed that PBMC mice displayed colons with shorter (*P* < 0.0001) and disorganized crypts (Figure 6, B and C) with increased apoptosis (Figure 6D), as compared with mice that also received DP8α Tregs. This appears particularly relevant, as apoptosis is used in the clinic to diagnose intestinal GvHD. Colons from both groups further differed in terms of blood vessel dilation and mucus production. Blood vessels appeared dilated in mice that received only PBMCs (Figure 6, B, D–F, and H), as compared with either conditioned NSG mice or PBMC+DP8α mice, likely due to massive human cell infiltration and/or local inflammation in PBMC mice. Interestingly, colons from mice treated with DP8α Tregs seemed to display an increased production/secretion of Alcian blue–stained mucus, as compared with PBMC mice (*P* = 0.0028) or even conditioned mice (*P* = 0.0014) (Figure 6, F and G). Additionally, staining of specific immune cell subsets confirmed flow cytometry results, i.e., infiltrates, mostly comprising human T cells (Supplemental Figure 9), were significantly lower in mice that received DP8α Tregs, as compared with PBMC mice. Indeed, hCD4^+^ T cells represented approximatively 17% and 12.5% of total cells in PBMC and PBMC+DP8α mice, respectively (*P* = 0.0092) (Figure 6, H and I). It is noteworthy that this difference appears even more relevant when the repeated injections of hCD4-expressing DP8α Treg cells are taken into consideration, while the PBMC group only received 1 injection of human PBMCs, comprising only a CD4^+^ cell fraction. Similar results were found regarding hCD8^+^ cells, which represented 8.2% and 5.5% of total cells in PBMC and PBMC+DP8α mice, respectively (*P* = 0.0052) (Figure 6, H and I).

DP8α Tregs therefore limited xeno-GvHD–related colonic inflammation, apoptosis, and human T cell infiltration, thereby protecting colon integrity possibly, in part, through heightened mucus production/secretion, in this model. Altogether, these results strongly support the hypothesis that CD73^+^ DP8α Tregs also control inflammation at a systemic level, in this model, without impairing human cell engraftment.

In accordance with our observations in patients, these mice data reinforce the fact that CD73-expressing DP8α Tregs could be pivotal to warrant aGvHD protection. Directly testing CD73’s role through either blocking or inhibiting hCD73 in such a model, while appealing, would unfortunately be uninterpretable, as CD73 is also expressed on other cell subsets in the injected PBMCs, including several T and B cell subsets. Additionally, CRISPR/Cas9-edited clones to abolish CD73 expression in these cells proved to be unfeasible, at least in our hands, as edited DP8α Treg clones failed to expand sufficiently to perform those experiments. We therefore addressed this question, as described below, using in vitro studies.

### DP8α Treg suppressive activity depends on CD73.

Based on the patients’ data (Figures 1 and 2) showing a link between defective CD73 expression on DP8α Tregs and aGvHD occurrence on d30 after transplantation, we aimed at directly assessing the role of CD73 on DP8α Treg suppressive function. Interestingly, we have previously demonstrated the role of the purinergic pathway in DP8α Treg suppressive function through CD39 inhibition experiments (31), but the direct role of CD73 in this context was yet to be determined.

**Figure 7:**
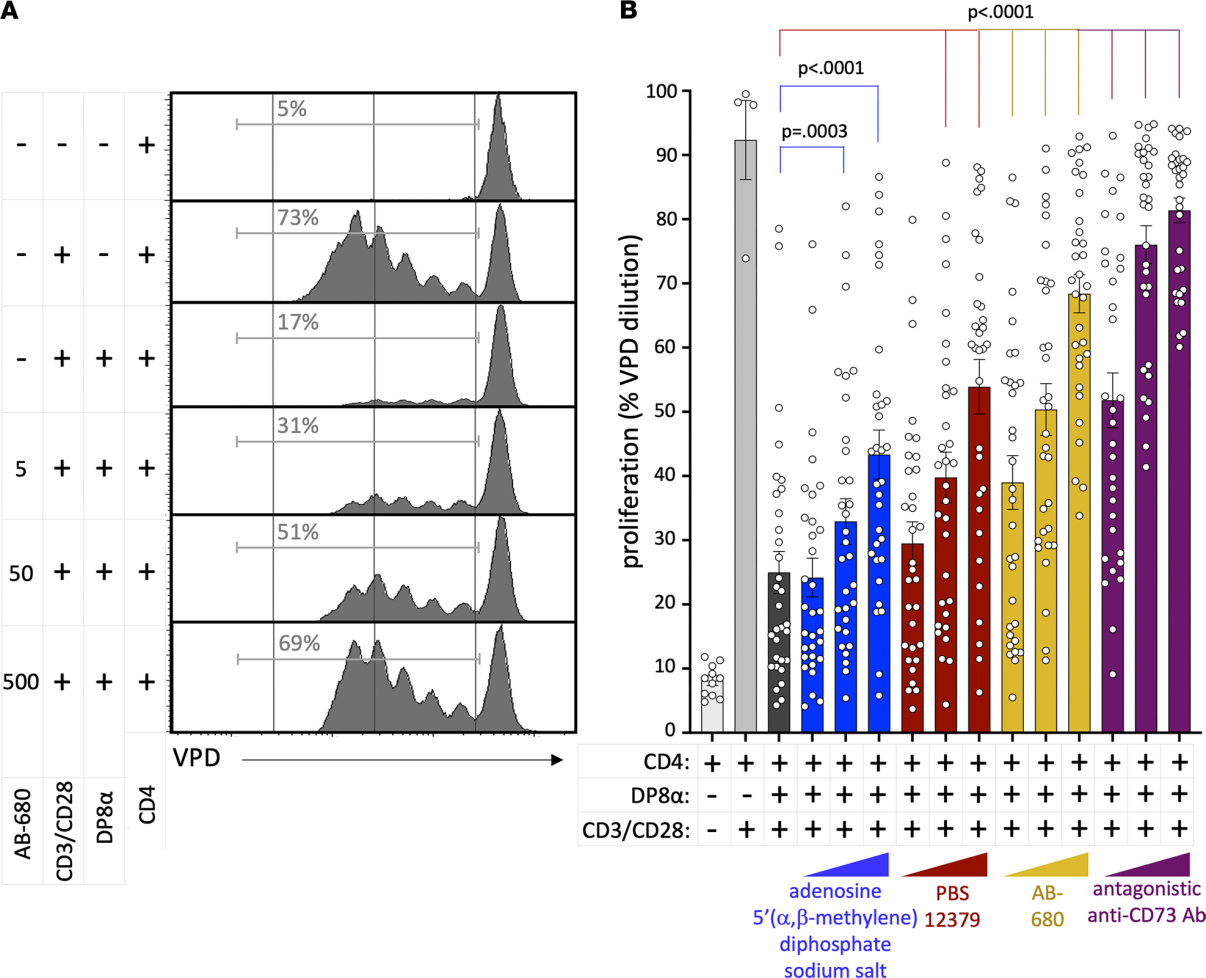
CD73 drives DP8α Treg suppressive activity. (**A** and **B**) Eight different DP8α Treg clones, whose CD73 expression is shown for a representative clone (Figure 2A), were all separately cocultured with sorted and VPD-stained CD4^+^ T cells derived from 4 different healthy donors (1:1 ratio) in the presence or absence of CD73 inhibitors (adenosine 5′-[α,β-methylene]diphosphate sodium salt at 2 mM, 20 mM, and 200 mM; PBS 12379 at 10 nM, 100 nM, and 1 mM; AB-680 at 5 nM, 50 nM, and 500 nM; blocking anti-CD73 antibody at 2 mM, 5 mM, and 20 mM). Proliferation was measured 5–6 days later as the percentage of VPD^lo^ CD4^+^ T cells. (**A**) A representative example for the coculture of a DP8α Treg clone with CD4^+^ T cells from 1 donor, with or without the CD73 inhibitor AB-680 at indicated concentrations, is shown. (**B**) The entire data set from this experiment is presented. One-way ANOVA with post hoc Dunnett’s multiple-comparison test was used to compare indicated conditions to the “no-treatment” data, corresponding to DP8α Treg inhibition of CD4^+^ T cell proliferation in the absence of any inhibitor. Results are represented as mean ± SEM.

To this end, we first used DP8α Treg clones derived from healthy individuals, whose CD73 expression is shown for a representative clone (Figure 4A). Strikingly, blocking CD73 on 8 DP8α Treg clones, prior to coculture, with escalating doses of either 3 distinct specific inhibitors or an antagonistic CD73-specific antibody, drastically impaired their ability to inhibit CD4^+^ T cell (purified from 4 different donors’ PBMCs) proliferation in a dose-dependent manner (Figure 7, A and B), establishing the key role for CD73 in DP8α Treg suppressive function and advocating for the pathophysiological relevance of their observed lack of CD73 in aGvHD patients 1 month after transplantation.

### Regulatory potential and host reactivity of donor-derived DP8α Tregs.

**Figure 8:**
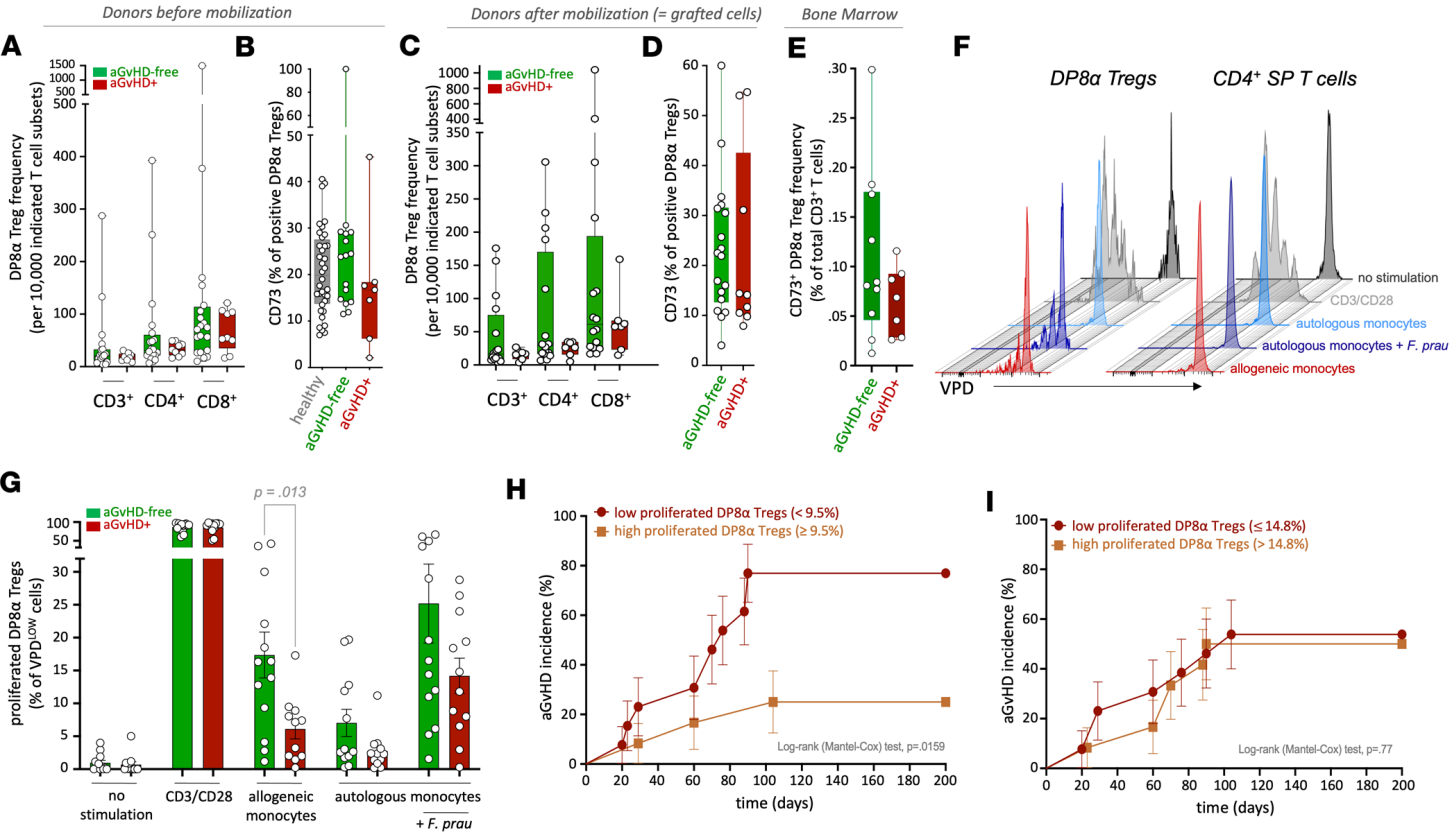
Regulatory potential and host reactivity of donor-derived DP8α Tregs tend to discriminate patients who will develop aGvHD from those who will not. (**A** and **B**) Circulating DP8α Treg frequency among indicated T cell subsets (**A**) and their CD73 expression (**B**) are shown for allograft donors before inducing mobilization with granulocyte colony–stimulating factor (G-CSF). (**C** and **D**) Circulating DP8α Treg frequency among indicated T cell subsets (**C**) and their CD73 expression (**D**) are shown on mobilized blood donors and thus corresponds to the actual grafted cells. (**E**) CD73^+^ DP8α Treg frequencies in bone marrow samples before transplantation. (**F** and **G**) CD4^+^ T cells, comprising DP8α Tregs, derived from HSCs’ donors were magnetically sorted and stained with 1 mM VPD before being cocultured in the presence of low-dose IL-2 (20 IU/mL) with either patient-derived magnetically sorted monocytes (ratio 1:1) (obtained before transplantation) previously loaded overnight or not with *F*. *prausnitzii* (ratio 1 monocyte:5 bacteria) or with magnetically sorted monocytes from the corresponding donors (ratio 1:1). Five days later, T cell proliferation of gated DP8α cells was measured through VDP dilution assessment by flow cytometry. Patients developing aGvHD or not (**G**; red and green, respectively) are shown. Results are represented as mean ± SEM. SP, single-positive. (**H** and **I**) Cumulative aGvHD incidence was plotted for low versus high allogeneic host-reactive DP8α cells (**H**) or *F*. *prausnitzii*–reactive DP8α cells (**I**), in all patients. Cutoff was determined using the median of host-reactive cells (= 9.5%, **H**) or *F*. *prausnitzii*–reactive cells (= 14.8%, **I**) among total DP8α cells. Log-rank (Mantel-Cox) test was used.

Our findings observed in patients’ blood (Figures 1 and 2) also raised the question whether CD73 deficiency preexisted on DP8α Tregs in the HSC donors. We therefore analyzed the frequencies of DP8α Tregs and their levels of CD39 and CD73 expression in the blood of donors collected both before and after mobilization. A nonsignificant trend toward a decrease for both DP8α Treg frequency (Figure 8A) and their CD73 expression (Figure 8B) was observed before mobilization in available donors whose grafts triggered aGvHD in patients, suggesting that donors with a reduced frequency of CD73^+^ DP8α Tregs could already harbor features making them prone to aGvHD development in recipients. Of note, only a few donors were available for this study (*n* = 7 aGvHD^+^, *n* = 16 aGvHD-free). A larger study would be needed to formally determine whether such DP8α Treg characteristics in donors really affect aGvHD incidence and hence represent a predictive tool. Besides, these trends were barely to no longer detectable after mobilization (Figure 8, C and D), showing that, if such biomarkers were indeed relevant, they should be measured before mobilization. We also collected, before transplantation, BM samples from 17 of the 62 patients tested. CD73^+^ DP8α Treg frequency tended to be higher in aGvHD-free patients (mean = 0.111% ± 0.0274%, *n* = 10), as compared with aGvHD patients (mean = 0.0664% ± 0.0130%, *n* = 7), not only showing that these cells were present in the BM compartment, but also suggesting that their frequency in BM could also be slightly decreased in aGvHD versus aGvHD-free patients (Figure 8E).

Host-reactive Tregs appear to be key in tolerance induction and maintenance in experimental transplant models, including GvHD models (39, 40). To start assessing whether donor-derived transplanted DP8α Tregs exhibit such an alloreactivity toward patient-derived antigens, we cocultured donor-derived CD4^+^ T cells stained with violet-proliferation dye 450 (VPD), comprising DP8α Tregs, with patient-derived monocytes (purified before transplantation). In addition, T cells were cocultured with purified autologous monocytes loaded with *F*. *prausnitzii* or not (as a negative control) to assess their reactivity to the bacterium. Twenty-five donor/recipient pairs were tested, among which 12 patients ended up developing aGvHD. Greater DP8α proliferative responses to host-derived antigens (*P* = 0.013) (Figure 8, F and G) and, to a lower extent, against *F*. *prausnitzii* in an autologous context, were observed for patients who did not develop aGvHD versus those who did (Figure 8, F and G). Indeed, proliferated donor-derived DP8α cells after coculture with host-derived allogeneic monocytes represented 17.4% ± 3.49% and 6.11% ± 1.51% as a mean, among total DP8α cells, for aGvHD-free patients and aGvHD patients, respectively (Figure 8G). Similarly, DP8α cells responded to the bacterium in an autologous context, with a mean of 25.2% ± 6.0% and 14.2% ± 2.69% for aGvHD-free patients and aGvHD patients, respectively (Figure 8G). Additionally, when patients were stratified according to their fraction of DP8α cells responding to host-derived alloantigens (cutoff: median = 9.5%), patients with low alloreactivity (<9.5%) developed aGvHD more frequently than patients with high alloreactivity (≥9.5%), with a hazard ratio of 4.42 and a 95% CI of 1.422 to 13.73 (Figure 8H). On the other hand, patients’ stratification based on their responses to *F*. *prausnitzii* did not show any difference between patients displaying DP8α cells with low versus high levels of responses against the bacterium (cutoff: median = 14.8%) (Figure 8I). These data strongly suggest that DP8α Tregs exert their protection against aGvHD (Figure 2, A–D), at least to a significant extent, through their reactivity to the host.

Furthermore, regarding only the first 15 patients transplanted (the 10 additional patients were transplanted too recently to be taken into consideration), despite an undeniable lack of hindsight due to the fact that these patients were transplanted just between 12 and 25 months prior to this study, patients who developed cGvHD (*n* = 4) at the time of this study displayed lower host-reactivity of their DP8α cells than patients who did not (*n* = 11) (Supplemental Figure 10A). A similar trend was observed regarding DP8α cell responses to *F*. *prausnitzii* (Supplemental Figure 10A). However, these 4 cGvHD patients had also undergone aGvHD; therefore, the response profile of DP8α cells could solely be due to the acute phase of the disease, as observed in the first cohort (Figure 2, F–J).

Of note, DP8α cells demonstrated a strong propensity to recognize alloantigens, even between matched donors and patients (Figure 8, F and G). Indeed, a 4-fold increase in the fraction of donor-derived cells responding to host-derived alloantigens was observed within the DP8α population, as compared with single-positive CD4^+^ T cells (Figure 8G and Supplemental Figure 10, A–C).

Altogether, these data suggest that DP8α Tregs are particularly prone to alloreactivity, making them well equipped to protect against GvHD. Moreover, the responses of donor-derived DP8α cells to *F*. *prausnitzii*, although to a lower extent than alloreactivity, supports the microbiota-dependent and colonic origin of these alloreactive Tregs.

Overall, these data represent robust corroborating evidence advocating for a key protective role of DP8α Tregs against aGvHD, likely in a CD73- and host-reactive-dependent manner, ostensibly without significantly increasing the risk of relapse.

## Discussion

*F*. *prausnitzii*–reactive DP8α Tregs exert potent suppressive functions in vitro, which depend on CD39 (31) and CD73 (Figure 7) enzymatic activities. In aGvHD patients, the presence of circulating CD73^+^ DP8α Tregs dramatically and specifically collapsed 1 month after transplantation (Figure 1, B–G, and Figure 2, A–D), as compared with (i) aGvHD-free patients, (ii) patients before transplantation, and (iii) healthy individuals. In contrast, cGvHD was not significantly linked with CD73^+^ DP8α Treg frequency at this relatively early d30 time point (Figure 2, F–J). Further studies remain to be conducted to determine whether the abundance of CD73^+^ DP8α Tregs at later stages could play a role in cGvHD development. Nevertheless, these data suggest that functionally competent DP8α Tregs could be, at least in part, involved in aGvHD protection, without affecting relapse odds (Figure 3, A–D). Additionally, no CD73^+^ DP8α Tregs were detected in patients who died of aGvHD, on d30 after transplantation (Figure 2, J–L, and Supplemental Figure 3), suggesting that the level of these cells could possibly correlate with aGvHD severity. Once again, a significantly higher number of patients would need to be studied to formally answer this question.

The fact that no difference was observed in CD39 expression on DP8α Tregs between aGvHD and aGvHD-free patients (Figure 1E), while CD73 expression on these cells was drastically reduced only in aGvHD patients (Figure 1B), is intriguing. Further studies, including in vivo experiments, similar to the one presented here, but with DP8α Tregs deficient in either CD39 or CD73, could possibly help understand the differential roles of these 2 enzymes. CD39 hydrolyzes extracellular ATP into ADP as well as ADP into AMP. CD73 can then cleave AMP into adenosine. Alternatively, AMP can be produced from ATP through CD203a (41). Furthermore, adenylate kinase-1 can also generate AMP from ADP (42). These 2 additional sources of AMP, in a CD39-independent manner, could possibly play a role in the fact that CD73 activity appears to represent a strategic step to regulate adenosine production. However, no explanation yet stands out regarding this discrepancy and the fact that CD73, but not CD39, seemingly represents a key relevant regulation element for the role of DP8α Tregs against aGvHD-related inflammation. Regardless, if the elevated expression of CD39 on DP8α Tregs in both patients’ groups suggests that ATP hydrolysis stays functional, the lack of CD73 on DP8α Tregs associated with aGvHD risk shows that the underlying mechanism essentially relies on an adenosine production defect. Additionally, it is worth mentioning the role of gut bacteria in adenosine metabolism. Indeed, some commensal bacteria seemingly either induce adenosine production or secrete inosine, the product of adenosine deamination, both of which signal through adenosine receptors and mediate antiinflammatory effects (13). Untangling the intimate interplay between gut bacteria and host-derived pathways, including microbiota-reactive CD73-expressing DP8α Tregs, to finely understand the regulation of adenosine receptor triggering, remains to be accomplished.

The NSG mouse model of acute xeno-GvHD clearly demonstrated the potent ability of DP8α Tregs to protect against induced GvHD (Figure 4) through inhibiting T cell infiltration and inflammation in most organs analyzed and preserving some microscopic/anatomic features of the colonic mucosa, such as length, crypt height, and mucus production. In the lungs, adenosine can promote mucus production/secretion through A1 adenosine receptor (A1AR) and A3AR triggering (13), suggesting that such a mechanism could occur in the gut, whereby CD73^+^ DP8α Tregs would stimulate mucus production via their ability to produce adenosine. Outstandingly, apoptotic areas were observed in the colon of PBMC mice, but not PBMC+DP8α mice (Figure 6D), which represents a relevant anatomopathological parameter since it is used in the clinic to diagnose intestinal GvHD. Of note, as the injected PBMCs and DP8α Tregs were not derived from the same donors, one can envisage the possibility of alloreactivity either from PBMC-derived CD4^+^ T cells against the DP8α Treg cells or from the DP8α Tregs against the PBMCs. Regarding the former hypothesis, if such a reaction had occurred in a substantial manner, the xeno-GvHD–related inflammation would therefore be expected to be exacerbated. No such worsening was evidently observed upon DP8α Treg injection (Figure 4, C–E), suggesting that this kind of alloreactivity did not play a significant role in this model. DP8α Treg cells could also exert some alloreactivity against PBMCs. These Tregs were TCR activated prior to injection, but such an alloreactivity could supposedly support maintaining their activation state and the associated disease protection effect. However, in vivo experiments consisting of a single injection of activated DP8α Tregs did not protect mice against xeno-GvHD (Supplemental Figure 11), refuting such a postulate. It is also worth stressing that DP8α cells used here are clonal, strongly limiting their likelihood of responding to alloantigens.

In future studies, we will take advantage of this preclinical model to further understand the role of DP8α Tregs in aGvHD by assessing (i) whether DP8α Treg infusion could also protect in a therapeutic setting, i.e., by injecting them 7–10 days after PBMC administration, rather than concomitantly; and (ii) the lowest Treg/PBMC ratio that could still exert a protective activity against xeno-GvHD, in this model. This would represent a critical point also to gain insight into future immunotherapy development endeavors.

Remarkably, the low fraction of DP8α Tregs expressing CD73 in aGvHD patients was observed regardless of their hematological disease, HLA matching, conditioning regimen, and prophylaxis treatment (Table 1), further supporting the major role of these cells in aGvHD prevention. Interestingly, DP8α responses against both alloantigens and, to a lower extent, *F*. *prausnitzii*–derived epitopes, tended to be higher in patients who ended up not developing aGvHD versus those who did (Figure 8, G–I), suggesting that DP8α cell stimulation, mainly through alloantigens, could help maintain this subset, which, in turn, could be involved in aGvHD protection. This trend was not observed regarding cGvHD even though additional patients with cGvHD, but not aGvHD, would be needed to confirm this hypothesis. It is worth stressing that this result does not preclude a role for DP8α Tregs in cGvHD prevention later after transplantation, i.e., at cGvHD onset. Importantly, the uniquely high frequencies of alloreactive cells among DP8α Tregs, as compared with single-positive CD4^+^ T cells, support the contribution of the former to HSCT tolerance (Figure 8, G and H, and Supplemental Figure 10). As most patients in this cohort had a chimerism above 90% on days 30, 60, and 100 (Supplemental Figure 5), DP8α Tregs after transplantation were mostly of donor origin in the vast majority of patients, supporting the role of these cells in aGvHD protection in a relevant manner. Determining whether DP8α Tregs cross-react between *F*. *prausnitzii* and some alloantigens or whether different DP8α clones respond to each antigen would need additional studies, for instance through DP8α Treg clone production.

Intestinal microbiota α diversity at engraftment predicts mortality in allo-HSCT recipients (14), demonstrating its key role in this pathological context. *F*. *prausnitzii* belongs to the Clostridia class. While no clear underlying mechanism has been identified, this group of organisms has been recognized to protect against GvHD (20–24, 43). DP8α Tregs, induced and activated by *F*. *prausnitzii*, could therefore represent, at least in part, how Clostridia might modulate GvHD.

Overall, while general high bacterial diversity has been associated with better HSCT outcomes, several individual bacterial taxa, including *Faecalibacterium*, have been associated with numerous clinical responses, such as better survival, decreased GvHD incidence, increased neutrophil engraftment, improved antitumor immunity, loss of intestinal toxicity, increased efficacy of immune checkpoint blockade, or has even been correlated with anti-CD19 CAR T cell immunotherapy (43–47). Whether some of the effects mentioned above, other than the ones described in our study, involve the DP8α Treg subset remains to be determined.

Altogether, our data demonstrated the protective role of a microbiota-reactive human cell subset, namely DP8α Tregs, against aGvHD, thus revealing a mechanism bridging the known influence of gut microbiota and CD73-mediated protection in this context. Moreover, our findings are in accordance with mouse studies showing that mouse CD73-deficient Tregs had an impaired ability to mitigate GvHD mortality, as compared with wild-type Tregs (12), likely through adenosine deficiency limiting Treg suppressive functions, such as those needed to dampen proinflammatory cytokine production by alloreactive T cells (12, 48).

Interestingly, other regulatory biomarkers, variably expressed by DP8α Tregs (34), were reported to play major roles in GvHD prevention such as granzymes A (49) and B (50) and CXCR3 (7, 51) and CCR5 (51, 52). A concurrent deregulation in the expression of such DP8α-related molecules could be necessary to trigger aGvHD and will need to be further investigated.

Altogether, these data support the notion that a CD73-dependent functional change in DP8α Tregs early after transplantation is, at least in part, involved in aGvHD occurrence. These results could therefore be used not only to eventually predict aGvHD risk, but also to develop innovative therapeutic strategies against aGvHD. Such treatments could be based on the infusion of DP8α Tregs exhibiting appropriate features. While the use of human canonical FoxP3^+^ Tregs had been envisaged, it holds several drawbacks, including their limited expansion yield, the fact that it could lead to preferential expansion of effector T cells (51, 53), and that a high FoxP3^+^ Treg/effector T cell ratio appears required (51). In contrast, DP8α Tregs can easily and reproducibly be expanded in vitro, while keeping potent suppressive properties (31), thus representing a promising candidate for Treg-based therapies. Moreover, administration of DP8α target antigens (*F*. *prausnitzii*–derived), in the form of peptides, proteins, or even bacteria/probiotics/prebiotics, could also represent a strategy to trigger their expansion/activation in vivo either directly or indirectly through the induction of tolerogenic dendritic cells (54) to limit GvHD-related inflammation.

## Methods

### Sex as a biological variable

Both male and female patients and healthy individuals were included and females represented 45% and 52%, respectively (see Table 1 for further information). Our animal study examined male and female mice, and similar findings are reported for both sexes.

### Immunostaining and antibodies

#### Flow cytometry.

PBMCs were isolated by Ficoll gradient centrifugation and stained for 45 minutes at 4°C in PBS/0.1% BSA with the following antibodies: anti-CD3 (clone UCHT1, BD), anti-CD4 (clone 13B8.2, Beckman Coulter), anti-CD8α (clone RPA-T8, BD), anti-CCR6 (clone G034E3, BioLegend), anti-CXCR6 (clone K041E5, BioLegend), anti-CD39 (clone A1, BioLegend), anti-CD73 (clone AD2, BioLegend), anti-hCD45 (clone H130, BioLegend) and anti-mCD45 (clone 30-F11, BioLegend). For mice, blood was treated with an hypotonic solution for red blood cell lysis prior to staining. Human chimerism (%) was calculated as follows: hCD45^+^/(hCD45^+^ + mCD45^+^) × 100.

Samples were analyzed using a BD LSR II flow cytometer and Diva (BD) and FlowJo software (FlowJo LLC).

#### Immunohistochemistry.

Intestines were harvested and measured after flushing the lumen with PBS. Each intestinal segment was rolled, snap-frozen in cooled isopentane after embedding in OCT compound (VWR Chemicals), and stored at –80°C. For morphologic analyses, hematoxylin/phloxine/saffron staining (HPS) was performed. Alcian blue staining of cryostat sections was used to detect mucus.

For immunohistochemistry, cryostat sections (5 μm) were fixed in acetone for 10 minutes at 4°C. After blocking with 2.5% (v/v) horse serum (Vector Labs) for 20 minutes, sections were incubated at room temperature for 60 minutes with primary antibodies against hCD4 (clone EPR6855, Abcam), hCD8α (clone CAL67, Abcam), hCD20 (ab244336, Abcam), hCD11b (clone EP1345Y, Abcam), hCD68 (clone EPR20545, Abcam), and activated caspase-3 (clone Asp175, Cell Signaling Technology). Sections were then incubated with peroxidase-conjugated horse anti-mouse secondary antibodies (Vector Labs) for 30 minutes at room temperature. Staining was developed with diaminobenzidine (Vector Labs) for 3 minutes, counterstained with Mayer’s hematoxylin, and mounted with xylene-based media. Images for analyses were acquired as whole-slide images with a Nanozoomer 2.0 slide scanner (Hamamatsu Photonics).

#### Measurement of morphometric parameters and infiltrate analyses.

Measurements of crypt height were performed on HPS-stained sections using QuPath software (55). Five representative regions of interest (ROIs) of colon sections harboring well-oriented crypts were randomly selected from each mouse. Sections containing less than 3–5 well-oriented villi were excluded from the analysis. Vessel diameter measurements were similarly performed (5 ROIs/mouse). For mucus quantification, blue-stained areas corresponding to secreted mucus and mucus-producing goblet cells, and total crypt area were measured using automatic structure recognition by deep learning (QuPath). Four representative ROIs were selected from each mouse.

Immunostainings of hCD4 or hCD8α markers were semiquantified using QuPath. Five ROIs were defined from each mouse and “positive cell detection” functions were used for detection.

### Inhibition assays

CD4^+^ cells derived from healthy donors’ PBMCs were magnetically sorted (Miltenyi Biotec) and stained with 1 mM VPD (BD Biosciences) before being cocultured with DP8α Treg clones (1:1 ratio). T cells were activated using anti-CD3/anti-CD28 beads (Gibco) with or without 3 distinct CD73 inhibitors: adenosine 5′-(α,β-methylene)diphosphate sodium salt (Tocris), PBS 12379 (Tocris), AB-680 (Clinisciences), or an anti-CD73 blocking antibody (BioLegend, clone AD2), as indicated. Five to 6 days later, VPD^+^ CD4^+^ T cells were stained and their proliferation (VPD^lo^ cells) assessed by flow cytometry.

#### Determination of T cell reactivity toward alloantigens and F. prausnitzii.

CD4^+^ cells, comprising DP8α Tregs, derived from HSC donors were magnetically sorted (Myltenyi Biotec) and stained with 1 mM VPD before being cocultured in the presence of low-dose IL-2 (20 IU/mL; Proleukine, Novartis) either with host-derived magnetically sorted CD14^+^ allogeneic monocytes (ratio 1:1; obtained before transplantation) or with similarly sorted donor-derived autologous monocytes (ratio 1:1) previously loaded overnight with *F*. *prausnitzii* (ratio, 1 monocyte:5 bacteria; obtained from the Commensal and Probiotic-Host Interactions Laboratory, UMR1319 Micalis, INRAe, Jouy-en-Josas, France) when indicated. Five days later, T cell proliferation of gated single-positive CD4^+^ T and DP8α cells was measured through VDP dilution assessment by flow cytometry.

### ELISA

Mouse sera were obtained from centrifugated clotted blood and tested for their human IL-10, IL-6, and TNF-α contents using the ELISA MaxDeluxe Set according to the manufacturer’s guidelines (BioLegend).

### Statistics

Basic statistical analyses were performed using GraphPad Prism version 10.1.1 with primarily Mann-Whitney (for single comparisons) or 1-way ANOVA tests and post hoc tests for multiple comparisons, as indicated in the figure legends. Multivariate logistic regression analyses (for Table 2) as well as cumulative incidence analyses (log-rank tests) were also carried out using GraphPad Prism version 10.1.1. A *P* value of less than 0.05 was considered statistically significant. Relapse, NRM, aGVHD, and cGVHD were calculated using the cumulative incidence while accounting for the presence of competing risks (56, 57), using R software version 4.2.2 (cuminc package; https://www.rdocumentation.org/packages/cmprsk/versions/2.2-12/topics/cuminc). NRM and relapse were analyzed as competing events, as well as deaths related to aGVHD and deaths unrelated to aGVHD. The competing risk for aGVHD and cGVHD was death or relapse.

### Study approval

#### Patients, donors, and healthy individuals.

All patients and their donors signed informed consent forms. All studies involving patients’ samples were approved by the ethical review board of Nantes University Hospital (06/15-CPP Ouest IV – Nantes). Healthy donors’ blood samples were provided by Nantes Blood Center (EFS, CPDL-PLER-2018-021). Samples were removed of all identifiers (see Table 1 for clinical information) and processed within 18 hours of collection.

#### Mice.

NSG mice (Charles River Laboratories) were bred by our humanized rodent platform in specific pathogen–free conditions (accreditation number C44-278). Eight- to 12-week-old mice (males and females) were x-ray irradiated at 1.5 Gy on d0, 6 hours prior to i.v. injection with 10 × 10^6^ human PBMCs from healthy individuals. When indicated, mice also received 30 × 10^6^ clonal DP8α Tregs activated 2 days before using a CD3/CD28 nanomatrix (TransAct, Miltenyi Biotec). This study was carried out according to the authorizations from the French Ministry of Research, APAFIS nos. 29259 and 33312.

### Data availability

Data are available in the Supporting Data Values XLS file, or from the corresponding author upon request.

## Author contributions

EG, FJ, and FA conceptualized the study. EG, PC, FH, AJ, CV, MAV, and FJ developed methodology. PC, TG, PP, AG, ALB, MJ, and VL procured human samples. MJ performed Fine-Gray statistical analyses. EG, FH, CV, VD, SL, MV, MDS, MM, AP, and MAV performed experiments. EG and FH generated figures. EG, FJ, FA, and PC acquired funding. EG, FJ, and FA supervised the study. EG wrote the original draft of the manuscript, which was reviewed and edited by EG, FJ, FA, FH, PC, and MJ.

## Supplementary Material

Supplemental data

Supporting data values

## Figures and Tables

**Table 1:**
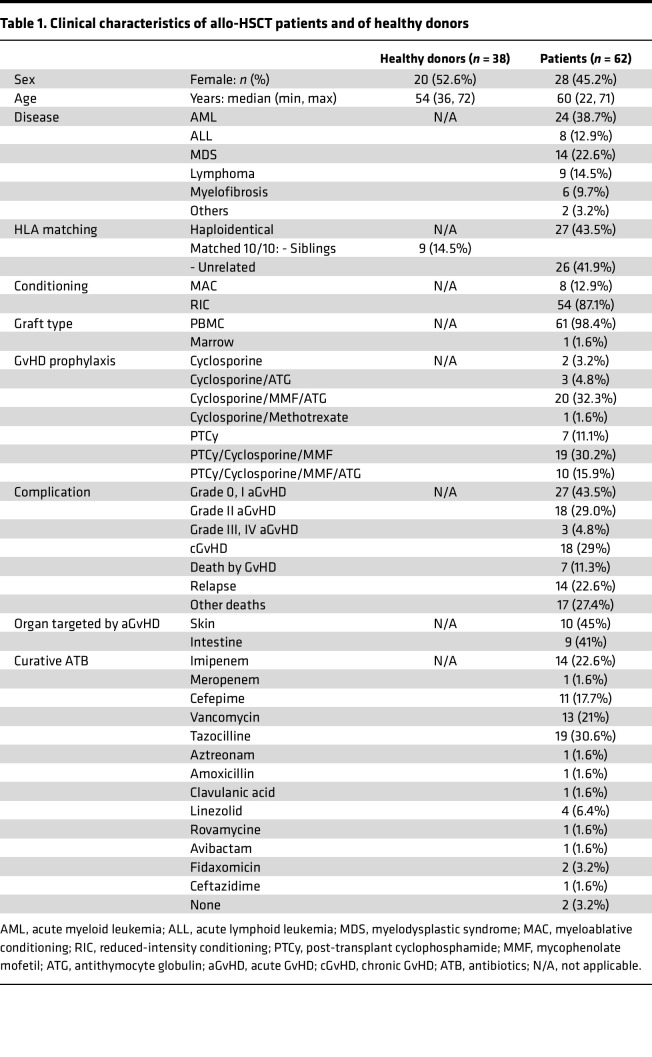
Clinical characteristics of allo-HSCT patients and of healthy donors

**Table 2:**
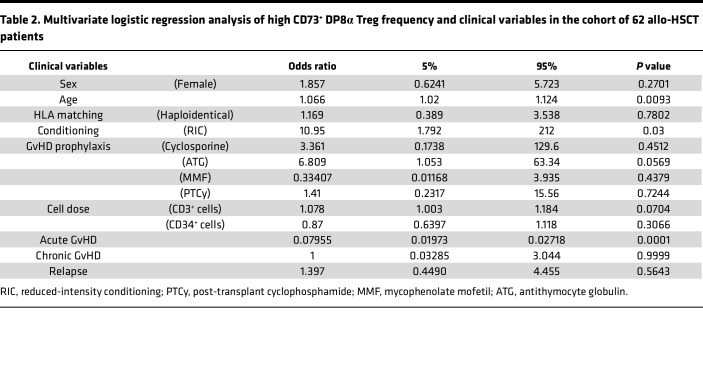
Multivariate logistic regression analysis of high CD73^+^ DP8α Treg frequency and clinical variables in the cohort of 62 allo-HSCT patients

## Version 1 08/01/2024

In-Press Preview

## Version 2 09/24/2024

Electronic publication
