# Supplementary material for: Human gut microbiota–reactive DP8α Tregs prevent acute graft-versus-host disease in a CD73-dependent manner
Source: JCI Insight. 2024 Aug 1;9(18):e179458. doi: 10.1172/jci.insight.179458 (PMC11457850; doi:10.1172/jci.insight.179458)
Supplement: Supplemental data [file jciinsight-9-179458-s057.pdf]

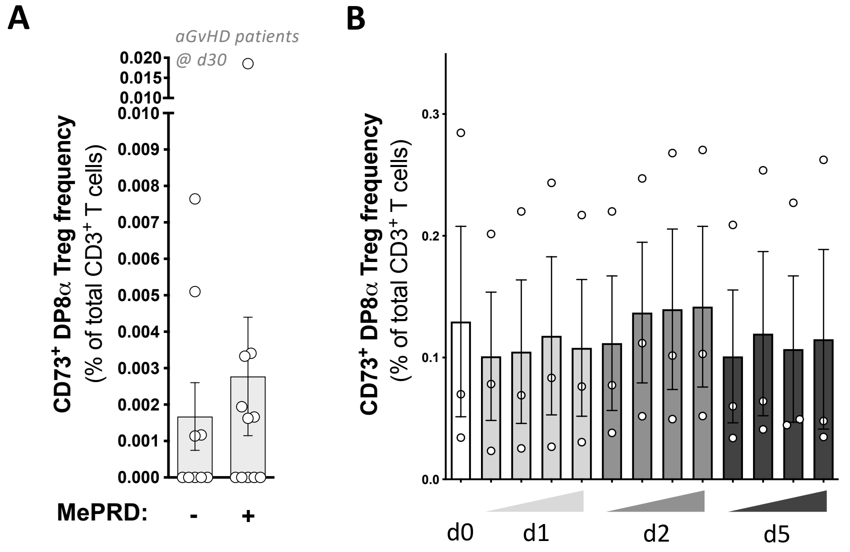

Figure S1. CD73⁺ DP8α Treg frequency is not affected by patients' corticotherapy. ***A***. CD73⁺ DP8α Treg frequency in aGvHD patients was determined as described in Fig.1 in patients who developed aGvHD after d30 analysis, none of whom had received corticoids (MePRD, methylprednisolone) yet, and in patients who developed aGvHD before d30 and therefore had received corticoids before d30 analysis. A Mann-Whitney test was performed; results are shown as mean+/-sem. ***B***. PBMCs from healthy individuals were treated *in vitro* with either the vehicle control, 0.2μM, 2μM or 5μM MePRD corticoid administered to aGvHD+ patients. CD73⁺ DP8α Treg frequency is shown at d0 (untreated), d1, d2 and d5 after treatment. One-way ANOVA (Kruskal-Wallis tests), followed by Dunn's multiple comparison tests to obtained adjusted p-values were used. Results are represented as means +/- sem.

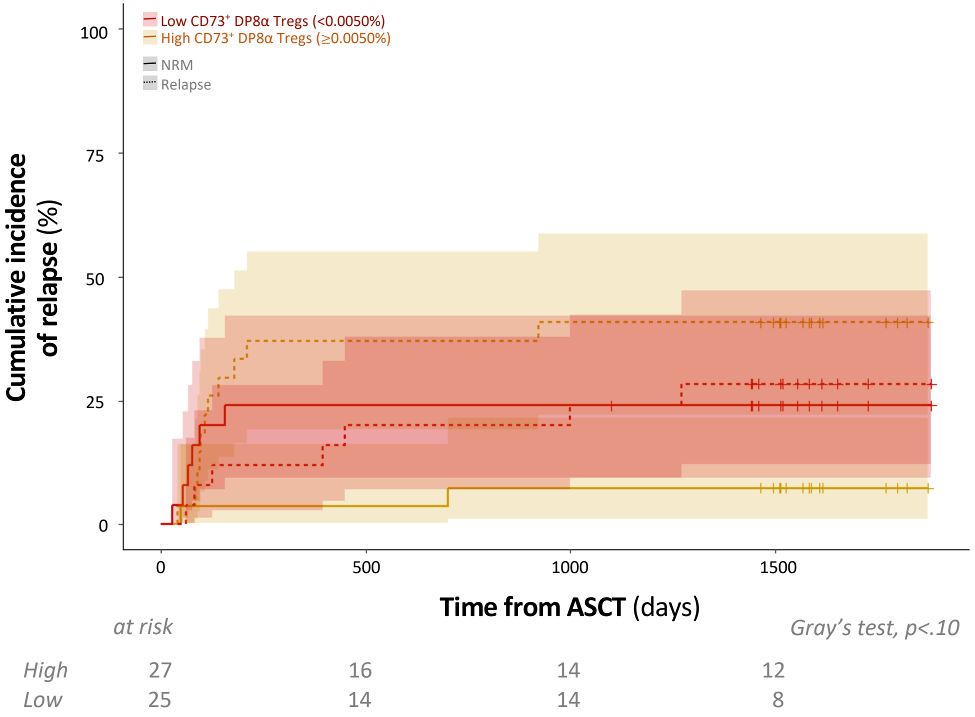

Group	Characteristics	3-year incidences	p-value
NRM	Group		0.10
	Low CD73⁺ DP8α Tregs (<0.0050%)	24% (9.5%, 42%)	
	High CD73⁺ DP8α Tregs (≥0.0050%)	7.4% (1.2%, 21%)	
Relapse	Group		0.3
	Low CD73⁺ DP8α Tregs (<0.0050%)	24% (9.4%, 42%)	
	High CD73⁺ DP8α Tregs (≥0.0050%)	41% (22%, 59%)	

Figure S2. Cumulative relapse and non-relapse mortality incidences depending on $CD73^+$ DP8α Treg frequency. Blood samples from healthy individuals (HD) and from patients with hematological malignancies were collected at 1-month post-transplantation. Samples were analyzed by flow cytometry to assess $CD3^+/CD4^+/CD8\alpha^{LOW}/CCR6^+/CXCR6^+/CD73^+$ DP8α Treg frequency among total $CD3^+$ T cells. The median (= 0.0050% of total T cells) was used as a cut off to constitute 'low' *versus* 'high' groups. Relapse (NRM as a competitive risk) and non-relapse mortality incidences are plotted for both 'low' and 'high' $CD73^+$ DP8α Treg groups. The Fine and Gray method was used for statistical analyses.

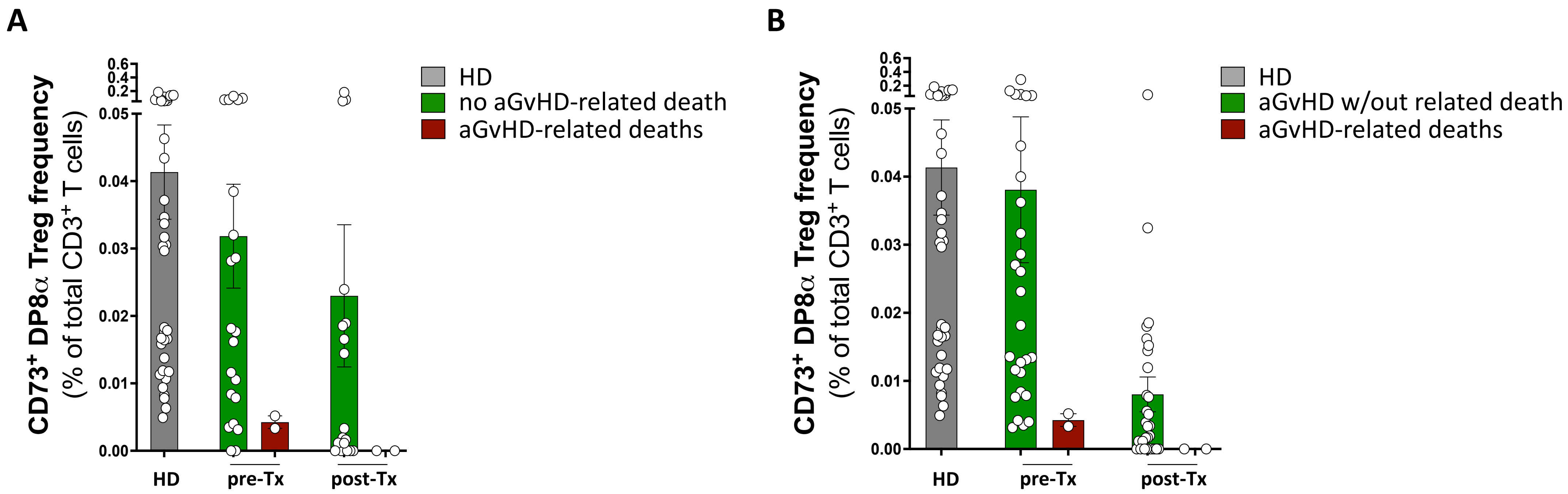

Figure S3. CD73-positive DP8α Treg frequency tend to be inversely associated with aGvHD-related death risk. Blood samples from healthy individuals (HD) and from patients with hematological malignancies were collected 7 days before receiving allo-HSCT (pre-Tx) and at 1-month post-transplantation (post-Tx). Samples were analyzed by flow cytometry to assess $CD3^+/CD4^+/CD8\alpha^{LOW}/CCR6^+/CXCR6^+/CD73^+$ DP8α Treg frequency among total $CD3^+$ T cells. **A.** Patients who died, irrespective of the cause, are represented. Patients who died from aGvHD are shown in red. Patients who died from other conditions are shown in green. ***B.*** All patients that developed aGvHD are represented. Patients who died from aGvHD (red) or not (green) were compared. Results are represented as means +/- sem.

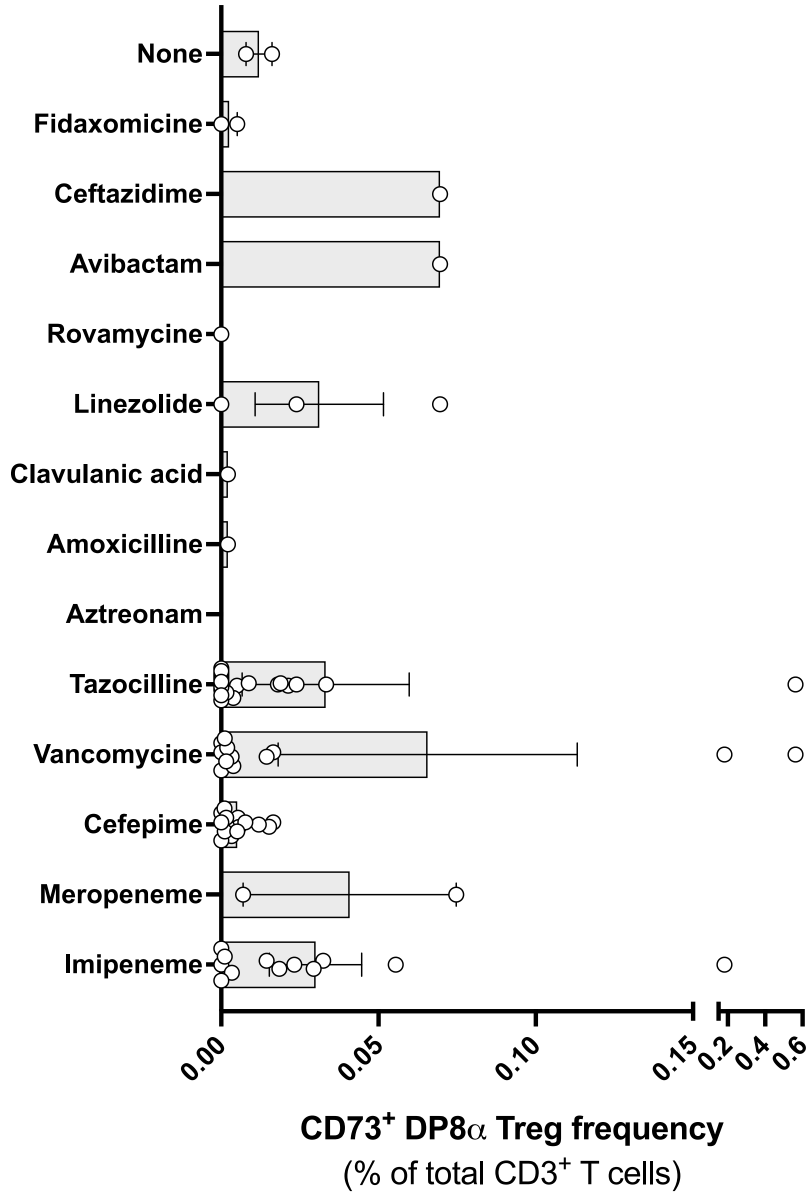

Figure S4. Patients' Antibiotic exposures. The frequency of CD73⁺ DP8α Tregs at 1-month post-transplantation in the 62 transplanted patients is represented for each antibiotic curatively used.

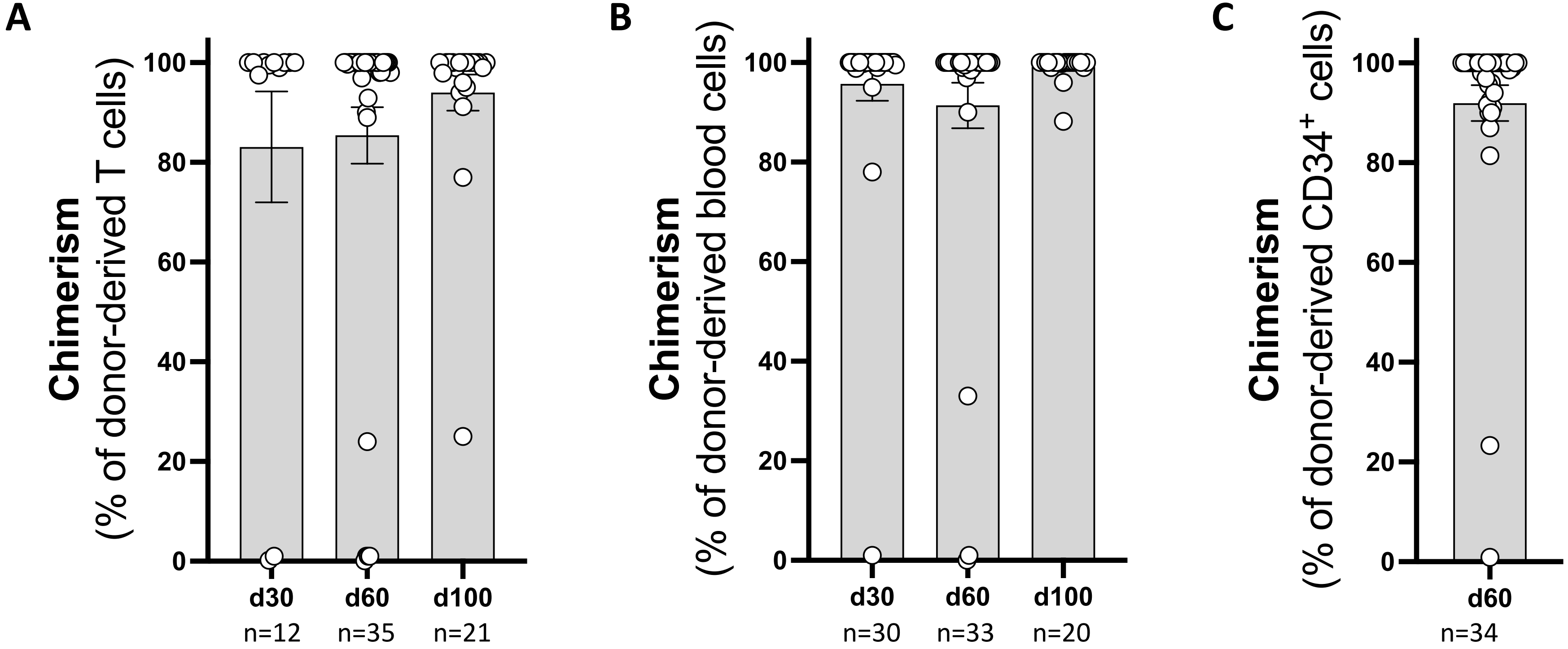

Figure S5. Patients' chimerism. Cell percentages of donor origin are shown at d30, d60 and d100 post-transplantation in patients when available, both in blood (**A.** for T cells or **B.** whole blood cells) and **C.** in bone marrow as a percentage of CD34-positive cells.

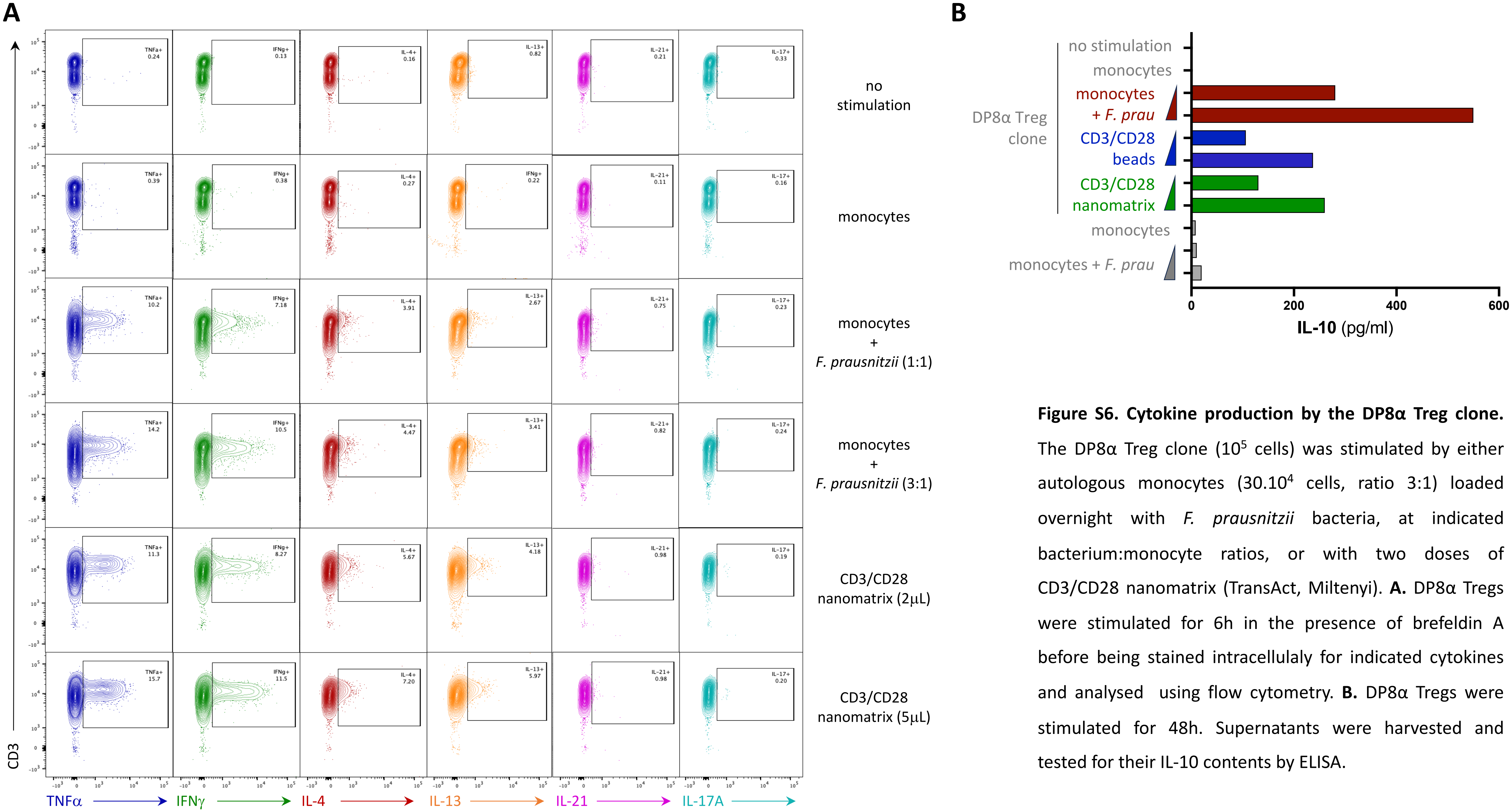

Figure S6. Cytokine production by the DP8α Treg clone. The DP8α Treg clone (10^5 cells) was stimulated by either autologous monocytes (30.10^4 cells, ratio 3:1) loaded overnight with *F. prausnitzii* bacteria, at indicated bacterium:monocyte ratios, or with two doses of CD3/CD28 nanomatrix (TransAct, Miltenyi). **A.** DP8α Tregs were stimulated for 6h in the presence of brefeldin A before being stained intracellulaly for indicated cytokines and analysed using flow cytometry. **B.** DP8α Tregs were stimulated for 48h. Supernatants were harvested and tested for their IL-10 contents by ELISA.

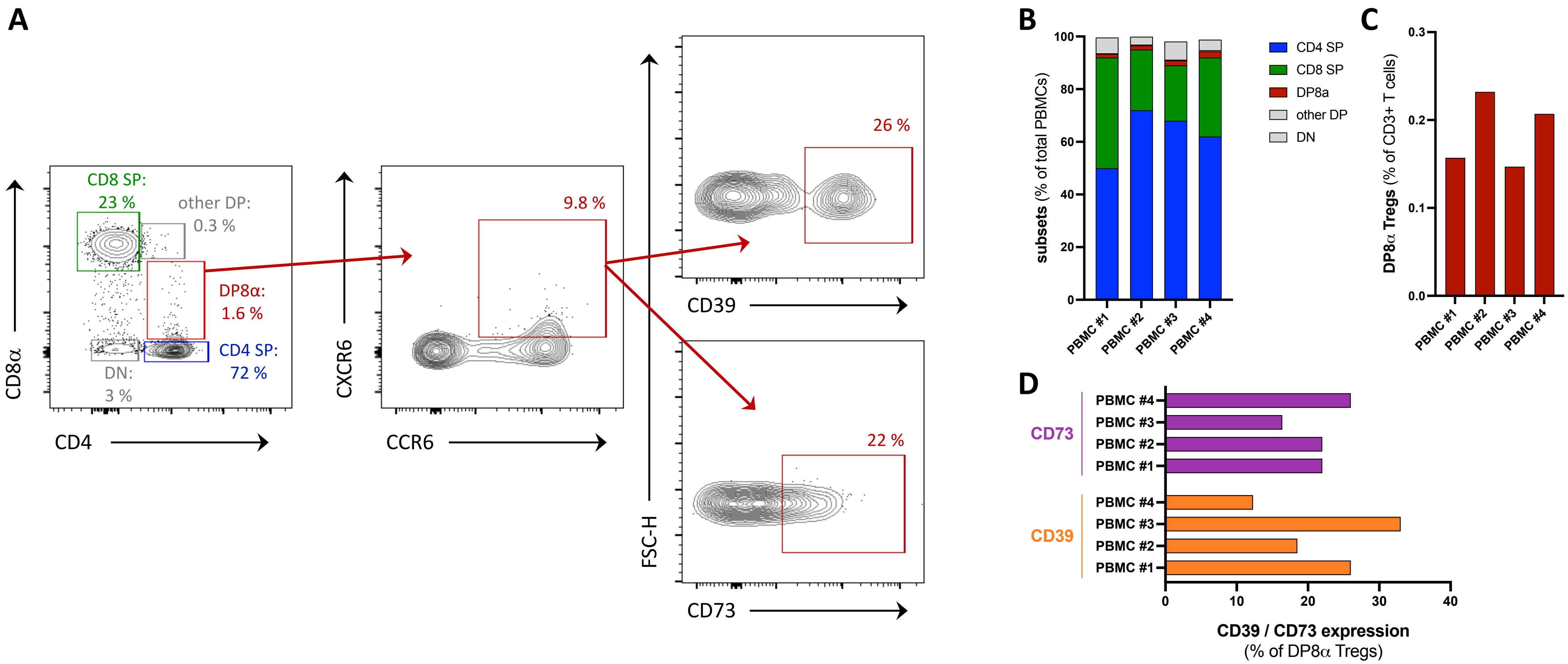

Figure S7. T cell subset composition of PBMCs injected to mice. As mentioned, this model is T-dependent and over 98% of PBMCs are composed of T cells, at least at d14 post-injection. **A.** Plots show T cell composition from one PBMCs donor pre-injection. CD4 SP (blue), CD8 SP (green), total DP8α (red) as well as other double-positive (DP) and double-negative (DN) cells (gray) are shown in the first plot. DP8α Tregs represent the CCR6^{+}/CXCR6^{+} fraction of total DP8α cells. The expression of CD39 and CD73 by the latter fraction is also shown for this PBMC donor. **B.** T cell composition is shown for the 4 PBMC donors. **C.** Percentages of DP8α Tregs (co-expressing CCR6 and CXCR6) among total CD3^{+} T cells for the 4 PBMC donors. **D.** Expression of CD39 (orange) and CD73 (purple) by DP8α Tregs derived from the 4 PBMC donors.

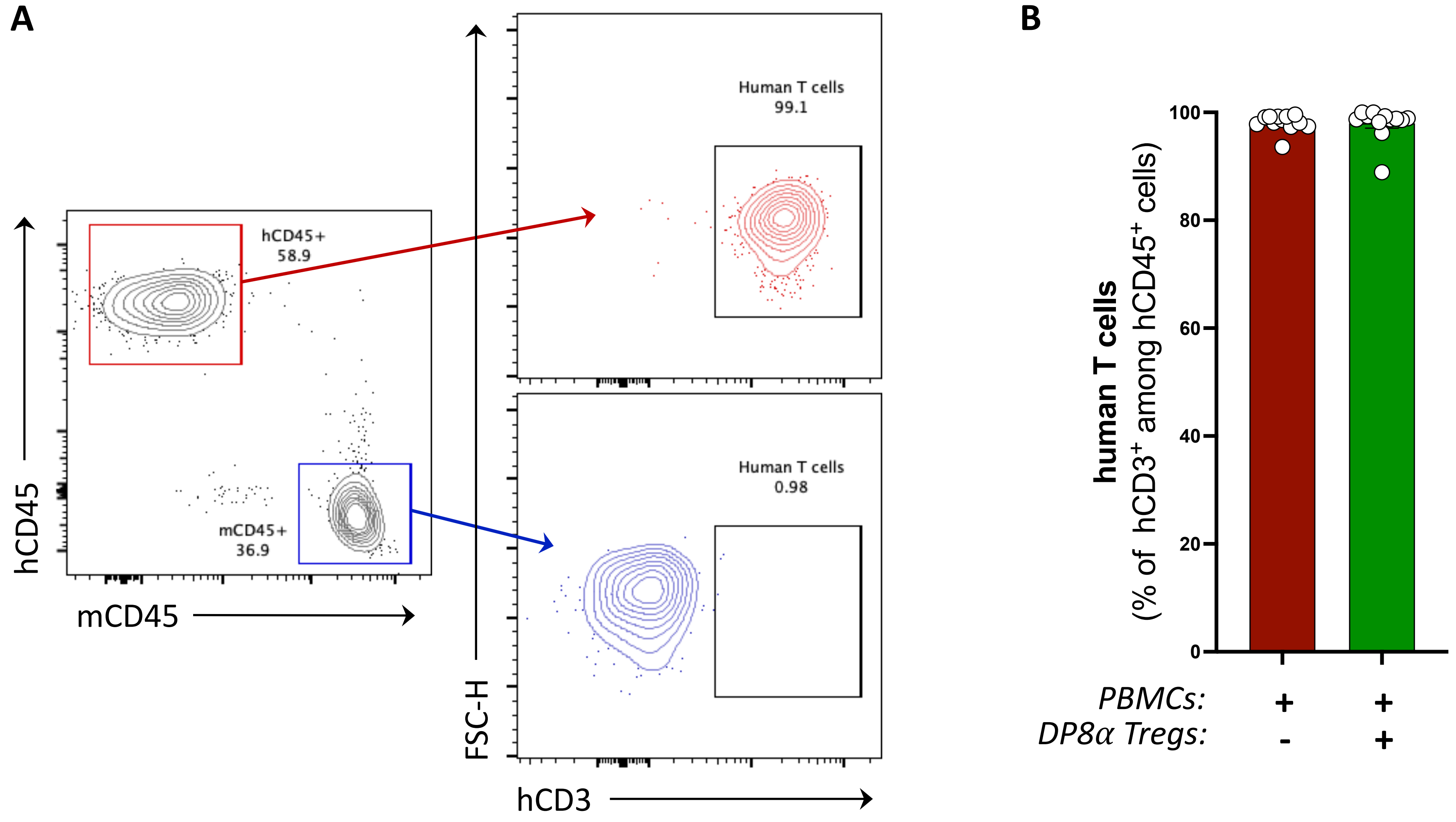

Figure S8. Human hCD45⁺ cells are mainly composed of T cells. Blood from all mice was drawn every week and stained for mCD45, hCD45 and hCD3 before flow cytometric analysis to follow chimerism. **A.** Contour plots from a representative example shows that the human CD45⁺ cells are virtually all hCD3⁺ T cells. **B.** All mice were similarly tested, showing that the human CD45⁺ cells are mostly composed of T cells at d14 in both groups of mice.

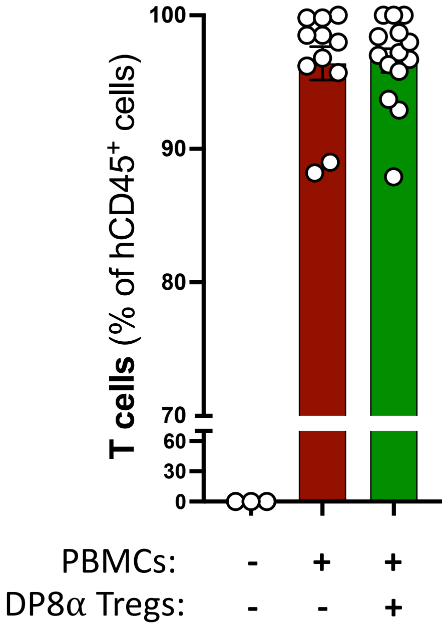

Figure S9. Human CD45^{+} cells infiltrating mouse colons are mainly T cells.
Colons were harvested at sacrifice, mashed and filtered before red blood cells were lysed as described in the Methods section. Cells were stained with hCD45, mCD45 and hCD3. T cells represented 96.41%±1.2 and 96.61%±0.9 in «PBMC» and «PBMC+DP8α» mice. Results are represented as means +/- sem.

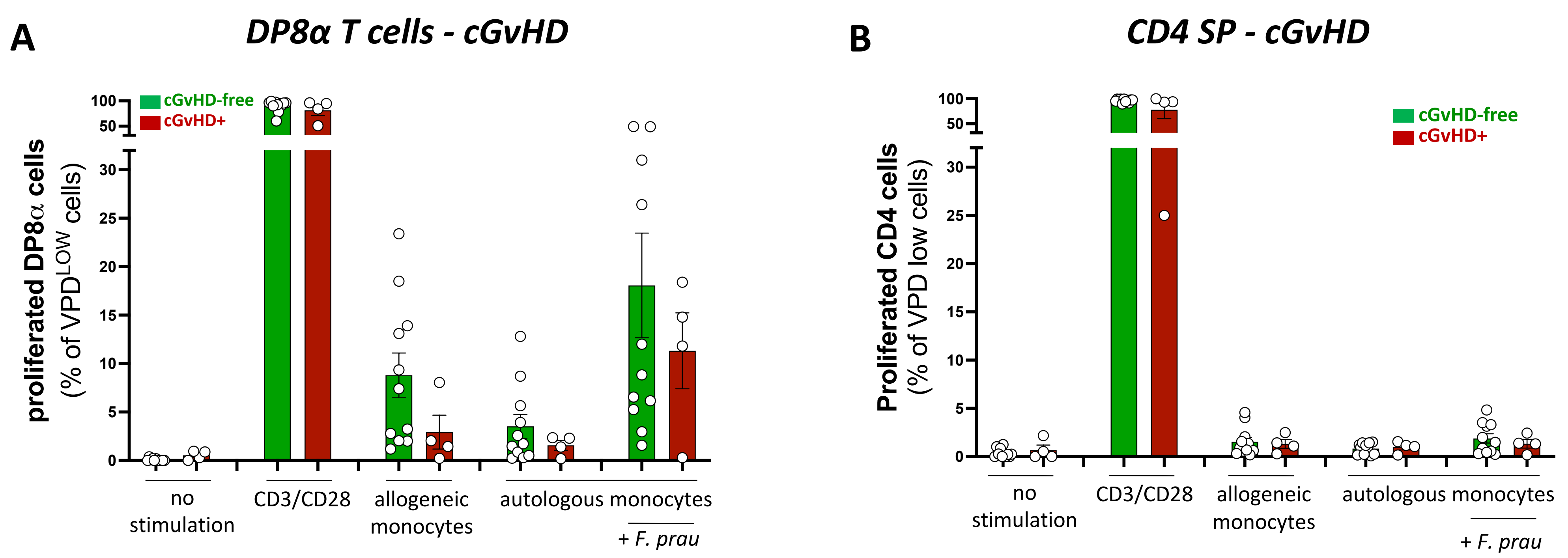

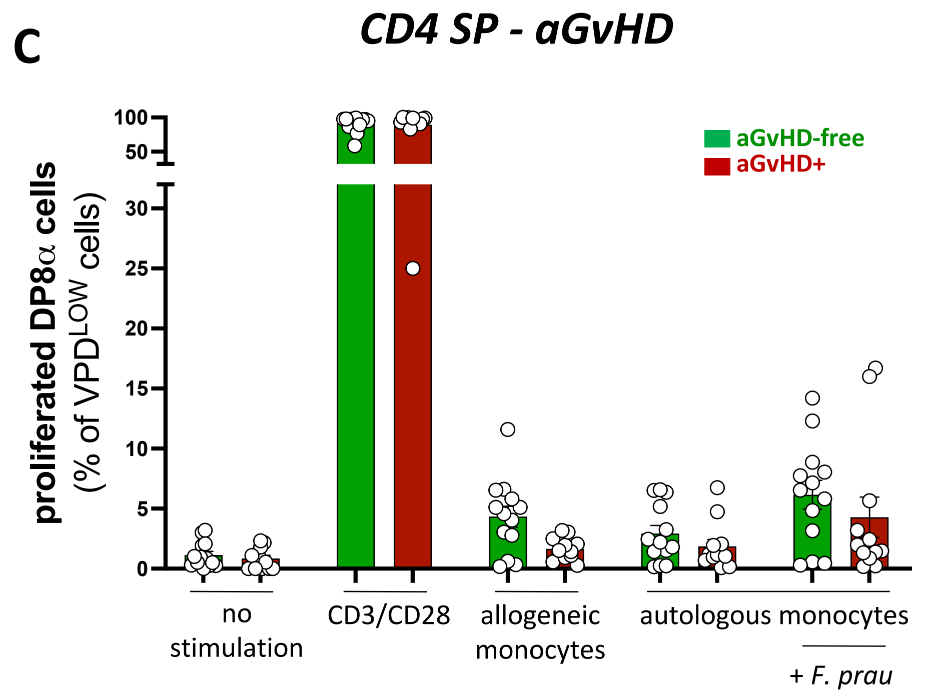

Figure S10. Host-reactivity of donor-derived CD4 SP and DP8α T cells from patients who will develop GvHD.
Total $CD4^+$ T cells derived from HSCs' donors were magnetically-sorted (Myltenyi) and stained with 1μM violet-proliferation dye (VPD) before being co-cultured, in the presence of low dose IL-2 (20 IU/ml) with either patient-derived magnetically sorted monocytes (ratio 1:1) (obtained before transplantation) previously loaded overnight or not with *F. prausnitzii* (ratio 1 monocyte:5 bacteria) or with magnetically-sorted monocytes from the corresponding donors (ratio 1:1). Five days later, T cell proliferation of gated subsets, i.e., single-positive $CD4^+$ T cells or DP8α T cells) was measured through VDP dilution assessment by flow cytometry. Patients developing cGvHD or not (**A.** and **B.** for SP CD4; red and green, respectively) and/or aGvHD (**C.** for DP8α T cells; red and green, respectively) are shown. Results are represented as means +/- sem.

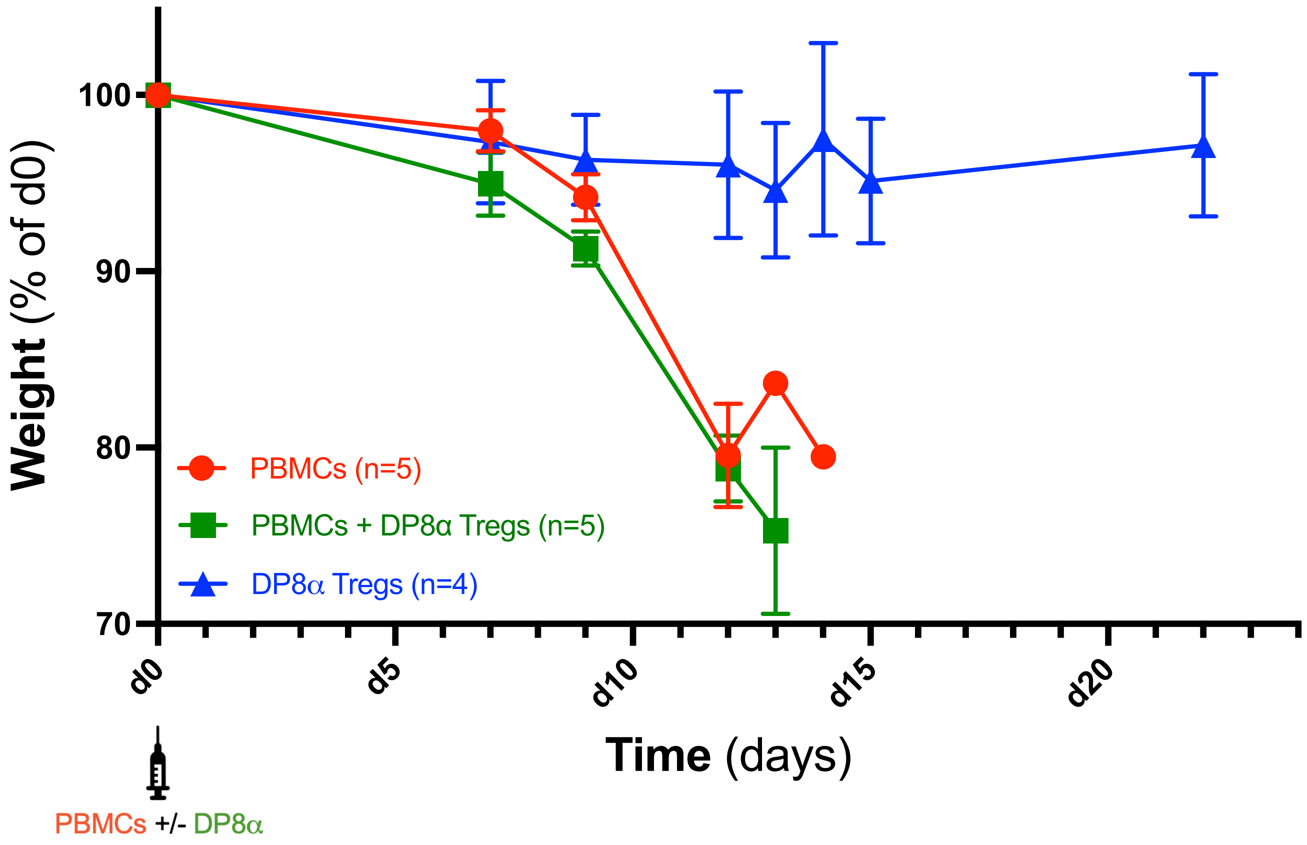

Figure S11. A single injection of human activated DP8α Tregs does not protect against xeno-GvHD *in vivo*. NSG mice were irradiated at 1.5gy at least 6h prior to be i.v. injected with 10 million freshly purified PBMCs from healthy individuals to induce xeno-GvHD (red). Another group of mice were also i.v. injected with 30 million DP8α Treg clonal cells (CD3/CD28-activated 48h prior infusion) at d0 (green). The third group of mice were injected with DP8α Tregs only (blue). Mice were weighted regularly, starting at d7. Mice had to be sacrificed when they lost 20% of their initial weight.
